# Electroactive nanoinjection platform for intracellular delivery and gene silencing

**DOI:** 10.1186/s12951-023-02056-1

**Published:** 2023-08-17

**Authors:** Ali-Reza Shokouhi, Yaping Chen, Hao Zhe Yoh, Takahide Murayama, Koukou Suu, Yasuhiro Morikawa, Jason Brenker, Tuncay Alan, Nicolas H. Voelcker, Roey Elnathan

**Affiliations:** 1 Monash Institute of Pharmaceutical Sciences, Monash University, 381 Royal Parade, Parkville, VIC 3052 Australia; 2 Melbourne Centre for Nanofabrication, Victorian Node of the Australian National Fabrication Facility, 151 Wellington Road, Clayton, VIC 3168 Australia; 3 Institute of Semiconductor and Electronics Technologies, ULVAC Inc, 1220-1 Suyama, Susono, Shizuoka 410-1231 Japan; 4 Department of Mechanical and Aerospace Engineering, Monash University, Wellington Rd, Clayton, VIC 3168 Australia; 5 INM-Leibniz Institute for New Materials, Campus D2 2, 66123 Saarbrücken, Germany; 6 Department of Materials Science and Engineering, Monash University, 22 Alliance Lane, Clayton, VIC 3168 Australia; 7 Faculty of Health, School of Medicine, Deakin University, Waurn Ponds, Melbourne, VIC 3216 Australia; 8 Institute for Frontier Materials, Deakin University, Geelong Waurn Ponds campus, Melbourne, VIC 3216 Australia; 9 The Institute for Mental and Physical Health and Clinical Translation, School of Medicine, Deakin University, Geelong Waurn Ponds Campus, Melbourne, VIC 3216 Australia

**Keywords:** Nanoelectroporation, Nanoinjection, Nanotube, Intracellular delivery, Transfection, Gene knockdown

## Abstract

**Background:**

Nanoinjection—the process of intracellular delivery using vertically configured nanostructures—is a physical route that efficiently negotiates the plasma membrane, with minimal perturbation and toxicity to the cells. Nanoinjection, as a physical membrane-disruption-mediated approach, overcomes challenges associated with conventional carrier-mediated approaches such as safety issues (with viral carriers), genotoxicity, limited packaging capacity, low levels of endosomal escape, and poor versatility for cell and cargo types. Yet, despite the implementation of nanoinjection tools and their assisted analogues in diverse cellular manipulations, there are still substantial challenges in harnessing these platforms to gain access into cell interiors with much greater precision without damaging the cell’s intricate structure. Here, we propose a non-viral, low-voltage, and reusable electroactive nanoinjection (ENI) platform based on vertically configured conductive nanotubes (NTs) that allows for rapid influx of targeted biomolecular cargos into the intracellular environment, and for successful gene silencing. The localization of electric fields at the tight interface between conductive NTs and the cell membrane drastically lowers the voltage required for cargo delivery into the cells, from kilovolts (for bulk electroporation) to only ≤ 10 V; this enhances the fine control over membrane disruption and mitigates the problem of high cell mortality experienced by conventional electroporation.

**Results:**

Through both theoretical simulations and experiments, we demonstrate the capability of the ENI platform to locally perforate GPE-86 mouse fibroblast cells and efficiently inject a diverse range of membrane-impermeable biomolecules with efficacy of 62.5% (antibody), 55.5% (mRNA), and 51.8% (plasmid DNA), with minimal impact on cells’ viability post nanoscale-EP (> 90%). We also show gene silencing through the delivery of siRNA that targets TRIOBP, yielding gene knockdown efficiency of 41.3%.

**Conclusions:**

We anticipate that our non-viral and low-voltage ENI platform is set to offer a new safe path to intracellular delivery with broader selection of cargo and cell types, and will open opportunities for advanced ex vivo cell engineering and gene silencing.

**Graphical abstract:**

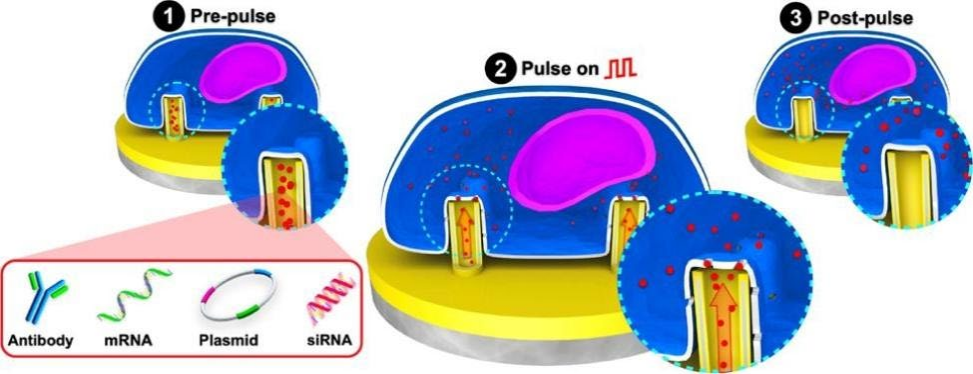

**Supplementary Information:**

The online version contains supplementary material available at 10.1186/s12951-023-02056-1.

## Background

Safely and efficiently accessing the intracellular environment is essential for effective intracellular delivery [1–4], cellular interrogation and manipulation [5–9], and cell engineering [10–12]—all pivotal to realizing biomedical innovation at the device–cell interface [13–16]. In particular, intracellular delivery of advanced biological effectors (e.g., gene-editing tools [17–19], nucleic acids [20–23], proteins [24–26], and nanoparticles [27–31]) is set to broaden the modality and cellular targets of therapeutic delivery for in vivo [32, 33] and ex vivo[34–37] gene therapy, CAR-T cell therapy [10, 38–43], and stem cell therapy [37, 44–47]. Successful cargo delivery depends on the ability to negotiate plasma membrane barrier through efficient and non-destructive approaches [13, 40]. Yet cell membrane presents a formidable challenge, limiting introduction of exogenous materials into the cell [48, 49].

Several approaches have been developed to increase the cell membrane’s permeability—either by exposing it to chemical agents [50, 51], viral vectors [52, 53], or cell-penetrating peptides [54, 55] (carrier-mediated), or by applying direct physical stress to the membrane (membrane-disruption-mediated) [3, 56]. Among these routes, the membrane-disruption-mediated approaches are at the forefront of intracellular delivery. These approaches can induce transient ‘holes’ in plasma membranes through mechanical [57–61], electrical (i.e., electroporation (EP)) [62–65], optical [66, 67], acoustic perturbation [68–70], or a combination of those effects [1, 56, 71–73]. These approaches offer higher level of flexibility in intracellular delivery, applicable across many cell and cargo types, which enables near-universal access; yet these approaches still suffer from significant limitations [1, 3, 74].

For example, conventional bulk EP (BEP) is a popular and simple approach that enables delivery of a wide range molecular cargos into different cell types via electrical stimulation [75, 76]. But lack of fine control over membrane disruption and spatial resolution, nonuniform electric field, high cell mortality due to use of high-voltage electric pulses (hundreds of Volts) and the subsequent Joule heating, and inconsistent delivery outcomes have made this approach less desirable [3, 77–79]. These drawbacks are now restricting the use of BEP in catalyzing therapeutic delivery for next-generation ex vivo cell-based therapy [78, 80].

Recent development in creating vertically configured nanostructure arrays, including nanowires [23, 25, 47, 81–88], nanostraws [89–97], and nanotubes [19, 98, 99] (NWs, NSs, and NTs) have resulted in the emergence of hybrid physical cellular nanoinjection platforms [71, 100]. The unique topological morphologies of these high-aspect-ratio structures allow for intracellular delivery of targeted cargos by generating highly localized stress on the cell membrane [101, 102]. Furthermore their assisted modalities—such as laser-assisted optoporation [96, 103], mechanical force applications [23, 58, 104, 105], and nanoelectroporation (nanoscale-EP) [37, 90–95, 97, 106–108] have further improved the delivery efficacy through minimal invasiveness and the capacity for achieving precise spatio-temporal resolution [2, 14, 35, 109].

For example, laser-assisted optoporation with plasmonic NTs have enabled targeted and transient plasma membrane disruption, achieving single-cell resolution through the ejection of hot-electrons via the 3D plasmonic nanoantennae at the NT’s rim [96]. But this route is often limited in scalability, due to the need for optical setups with a coherent laser source that can be complicated and expensive. In addition, the laser spot is typically scanned through the sample at a small region and slowly, further limiting the throughput [67, 110].

Applying mechanical forces (e.g., centrifugation, micromanipulators or pressing) offers a more straightforward approach to increase cellular adhesion and the capacity to remodel plasma membrane and stimulate endocytosis for cargo uptake [23, 81, 111, 112]. For instance, centrifugation conveniently applies a well-controlled, predictable, and reproducible force, but it needs to be optimized across different cell types [14, 104, 113]. Another key limitation of the mechanical routes is the lack of temporal control over membrane disruption [35].

Nanoscale-EP platforms overcome some of these shortcomings by inducing on-demand transient ‘holes’ in the cell membrane for a rapid and direct intracellular access [78, 114]. Such method offers superior performance over conventional BEP by inducing a highly localized and uniform electric field at the cell–nanostructure interface; this dramatically lowers the applied voltage threshold required for efficient EP, from hundreds to only tens of volts (20–40 V) [79, 80, 106]. However, most reported nanoscale-EP platforms have three inherent drawbacks: first, they suffer from prolonged and complicated fabrication routes [91, 107]; second they lack precise control over nanostructure topological parameters such as diameter, position, and spacing, which can lead to inconsistent delivery outcomes [115]; third, they require an integrated microfluidic reservoirs to deliver cargos into the cells during the nanoscale-EP [37, 90, 92, 97], which can complicate the platform’s fabrication and operation. To overcome some of those challenges, we introduce an electroactive nanoinjection (ENI) platform that offers four key advantages: (1) Fabrication: The precise control over specific topological parameters of the ENI platform increases precision over access and delivery by allowing modulation of the strength, localization, shape, and density of the induced electric fields. Critically, such programmable design and fabrication of the ENI platform allows for a non-destructive intracellular access with minimal cellular perturbation by dramatically lowering the applied voltage threshold to ≤ 10 V—significantly lower than conventional BEP [1, 78, 79]. (2) Versatility: Each NT consists of an inner central cavity, which offers the capacity to directly load diverse range of cargos such as proteins and nucleic acids. The ENI platform offers versatility in cargo delivery for targeted cell types. (3) High delivery efficiency (compared to BEP): The platform demonstrates high delivery efficiency across diverse cargo types, ensuring effective cargo delivery into targeted cells, with the added benefit of requiring 1000-fold lower cargo quantity and 20-fold lower voltage values compared to BEP. (4) Preservation of cell viability and cargo functionality: The ENI platform’s gentle (≤ 10 V) and localized treatment limits significant damage to the cells (> 90% cell viability) and the cargos post nanoscale-EP, evident by the expression of injected exogenous mRNA and pDNA, and the successful gene silencing caused by injected siRNA.

Here, we establish the ENI platform as a non-viral, low-voltage, and reusable tool that allows for precise nanoscale-EP with enhanced spatio-temporal resolution for intracellular delivery and ex vivo gene silencing. We demonstrate the ability to precisely engineer passive mechanical nanostructure cues with defined geometrical dimensions, in the form of vertically aligned silicon NTs (VA-SiNTs), and to transform them into their electroactive analogue. The engineered ENI platform offers the capacity to directly load targeted cargos into the NTs by exploiting their inner central cavity as reservoirs, eliminating the need for microfluidic channel integration during the nanoscale-EP. This significantly reduces cargo consumption, which is a limiting factor for microfluidic-based intracellular delivery systems [74]. The unique topography of these programmable and conductive NTs generates highly localized and uniform electric field, resulting in formation of transient pores in the membrane at the point of contact between the membrane and each conductive NT; this allows for influx of cargos from the NTs into the cell. Critically, the electric field that is emanating from the ENI platform dramatically lowers the required applied voltage, from hundreds to voltages ≤ 10 V, for efficient membrane poration with negligible cellular damage. Through both theoretical simulations and experiments, we demonstrate the ENI platform’s ability to electroporate GPE-86 mouse fibroblast cells and inject three membrane-impermeable cargos with relatively high efficiency (antibody: 62.5%, messenger RNA (mRNA): 55.5%, and plasmid DNA (pDNA): 51.8%), with minimal impact on cell viability post nanoscale-EP (> 90%). We also demonstrate gene silencing through delivery of small interfering RNA (siRNA), achieving 41.3% gene knockdown efficiency. The ability to efficiently deliver a broad range of exogenous materials—regardless of their type, size, and surface charge—is essential for advancing our fundamental understanding in designing biointerfaces, and for developing better approaches for cellular delivery.

## **Results and discussion**

### The ENI platform’s operation

**Fig. 1:**
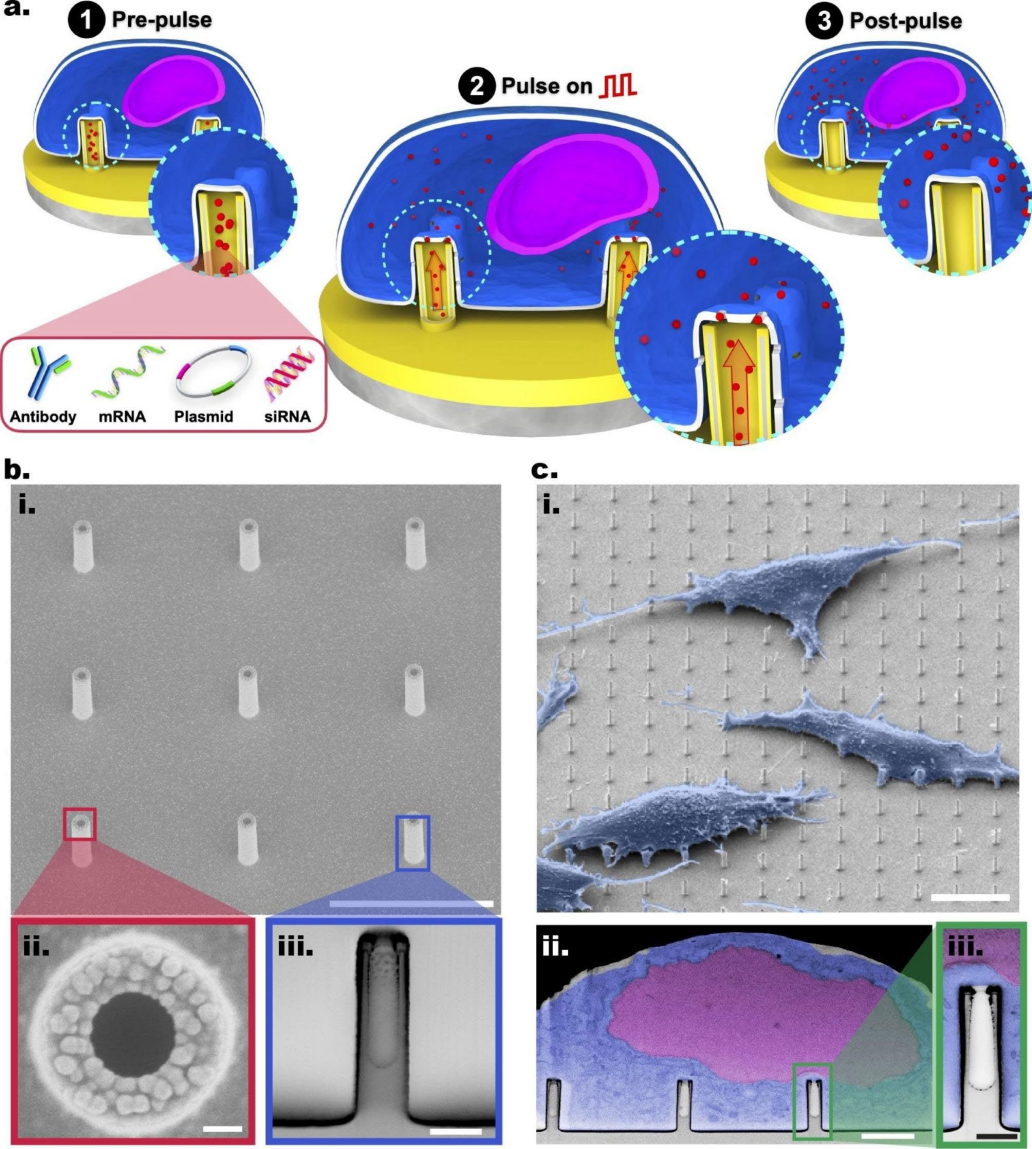
The ENI platform. **a** The steps of the ENI platform’s operation: The Au-coated NTs loaded with targeted cargo (stage 1); localized on-demand nanoscale-EP and the subsequent intracellular delivery (stage 2); and post nanoscale-EP membrane recovery (stage 3). **b** SEM images showing the Au-coated NTs at tilted zoom-out (i), top (ii), and cross-sectional (resin embedded) view (iii). Scale bars: 5 μm (i), 100 nm (ii), and 500 nm (iii). **c** False-colored SEM images showing the interfacial interactions between fibroblast GPE-86 cells and Au-coated NTs arrays (i) and the cross-sectional profile of NT–membrane interface (ii) with a zoom-in view (iii). Scale bars: 10 μm (i), 2 μm (ii), and 500 nm (iii)

The ENI platform consisted of arrays of Au-coated VA-SiNTs; a detailed fabrication process is described and illustrated in Supplementary Sect. 1.1 and Scheme S1, respectively (Additional file 1). The operation of the ENI platform consisted of three main stages (Fig. 1a). In stage 1 (pre-pulse), the NTs were loaded with cargos through drop-casting onto the pattern area (3 mm × 3 mm). To confine cargo access to only the pattern area and minimize reagent use, we placed a biocompatible polydimethylsiloxane (PDMS) enclosure onto the substrate with an opening consistent with the size of the pattern area (Additional file 1: Fig. S1). This enclosure allowed the solution containing cargos to be added onto the pattern area without any leakage. Post cargo loading, mouse embryonic fibroblast (GPE-86) cells were cultured onto the NTs with density of 0.1 × 10^6^ cells/mL for 3 h. The presence of the PDMS enclosure constrained the cells to only interface with the NTs. In stage 2 (pulse on), the samples containing cells were sandwiched between two biocompatible 3D-printed constructs (holder; discussed in Additional file 1: Supplementary Sect. 1.2). The holder was designed to house three samples in parallel and a Pt electrode. Once closed, the holder maintained the same distance (200 μm) between the samples and the Pt electrode during the nanoscale-EP. The holder also created a tight seal around each ENI platform once closed, limiting media/buffer leakage during the nanoscale-EP process (Additional file 1: Fig. S2). To induce localized membrane perforation, a train of low-voltage square pulses (≤ 10 V; 20 Hz; 400 µs; 600 cycles) were applied to the system, leading to formation of transient pores on the plasma membrane at the NT–cell interface, and subsequent influx of cargos from the NTs into the cells. In stage 3 (post-pulse), the cells were rested for 30 min on the NTs at 37 ºC and 5% CO_2_. The complete ENI platform’s workflow process is described and illustrated in Supplementary Sect. 1.3 and Scheme S2, respectively (Additional file 1).

At the core of the ENI platform is the Au-coated VA-SiNT arrays (Fig. 1b, i), fabricated through electron beam lithography and dry etching. This fabrication approach allowed for precise engineering of the NT geometry [14, 19, 35]. The VA-SiNTs were transformed into their electroactive analogue by sputter coating a thin layer of Ti/Au onto the NTs; this allowed for a biocompatible and conductive platform [91]. We designed and fabricated our ENI platform with pre-defined geometry: inner/outer diameter 300/500 nm (Fig. 1b, ii), height 2 μm, and pitch 5 μm, evenly distributed within a 3 mm × 3 mm region. A cross-sectional profile of the NT (Fig. 1b, iii) was obtained though scanning electron microscopy (SEM) imaging after focused-ion beam (FIB) milling, which confirmed the presence of a central inner cavity (0.12 µm^3^) within each NT, allowing for the direct cargo loading through the 300 nm opening. In this study, the 3 mm × 3 mm region for the pattern area was chosen as proof-of-concept. Yet, this top-down approach can potentially be used to scale up the number of NTs, allowing more cells to be processed.

The Au-coated NTs were coated with poly-D-lysine (PDL) to increase surface hydrophilicity of the platform, allowing even spread of solution containing the cargos, as well as promoting cell attachment onto the Au-coated NTs after the UV sterilization process (Additional file 1: Fig. S3). Post cell seeding onto the NTs, through SEM imaging, we observed that most GPE-86 cells spread across the platform and form focal adhesions on NTs after only 3 h incubation (Fig. 1c, i). Using FIB-SEM, we further examined the cross-sectional profile of the NT–cell interface, and found the cell membrane closely wraps around the NTs (Fig. 1c, ii and iii). Such membrane entanglement is crucial for a successful nanoscale-EP, as tightly coupled and strengthened cellular interfacing can increase the efficacy of localized EP and the subsequent pore formation on the membrane, and influx of exogenous cargos into cells [1, 91, 97, 110].

### Theoretical modeling of the electric field distribution across ENI platform

**Fig. 2:**
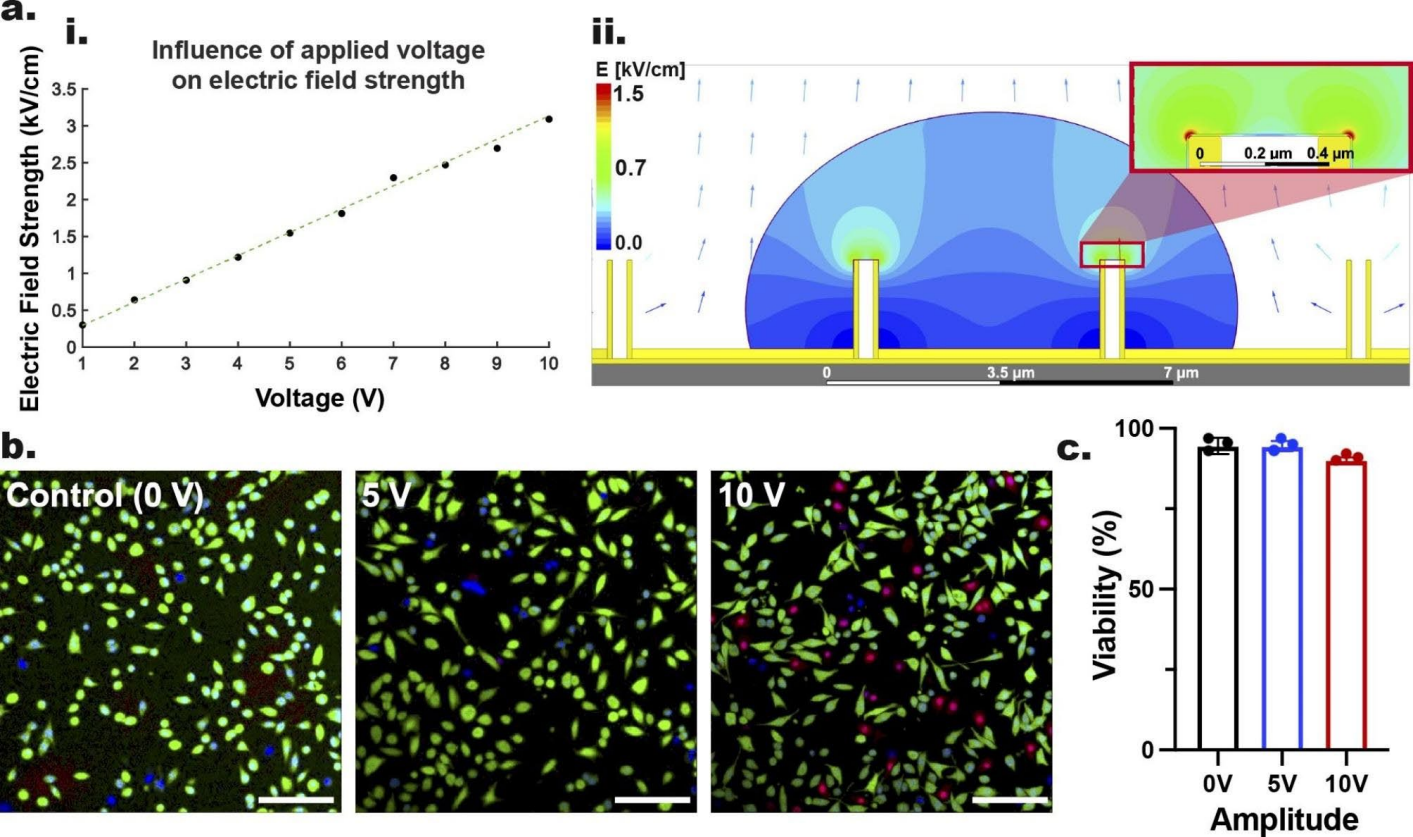
The electric field distribution across the Au-coated NTs during nanoscale EP, and viability of GPE-86 cells post nanoscale-EP. **a** Simulations showing the electric field strength at different applied voltage values ranging from 1 to 10 V (i) and its distribution across Au-coated NTs during application of 5 V pulses (ii). **b** Confocal microscopy images of GPE-86 cells before and after nanoscale-EP are compared using 0, 5 and 10 V amplitude values. Scale bars: 100 μm. **c** Quantification of the percentage of live cells post nanoscale-EP. Error bars indicate ± SDs, n = 3, one-way ANOVA.

To understand the mechanism of electric field localization at the NT–cell interface, we investigated the electric fields distribution across the ENI platform through ANSYS (2020 R1, simulation software) using the Maxwell 2D module. For these simulations, a 2D model of NTs with 2 μm height and 100 nm thick walls was created. The computational domain was set to be a region (20 μm wide) that included four NTs evenly spaced 5 μm apart. Cells were assumed to conform to the shape of the platform and be in close contact with the conductive NTs, avoiding presence of media between cell membrane and the NTs. Using ANSYS default material properties, the bottom planar surface and the reference electrodes were set as Au and Pt, respectively. Based on experimental setup, the distance between Au-coated NTs and Pt electrode was set to be 200 μm (Additional file 1: Fig. S4a). A mesh convergence study was performed considering the maximum electric field strength at the rim of the NTs (the region of highest electric field localization). This resulted in a mesh model containing approximately 200,000 elements with additional refinement in the cell membrane region around the top of the NTs (Additional file 1: Fig. S4b, i–iv). Using the simulated model, the maximum electric field strength was calculated for range of applied voltages: 1–10 V (Fig. 2a, i), which suggested that the electric field strength experienced by the cell membrane is linearly correlated to the applied voltage. Figure 2a, ii shows distribution of electric field lines through the ENI platform during the nanoscale-EP. At 5 V applied voltage, the concentration of electric fields on NTs’ rim indicated a strength equivalent to approximately 1.5 kV/cm (average at the NT’s rim). To understand this edge-effect, a 2D plot of maximum electric field strength across the NT’s rim was obtained, which suggested the occurrence of highest level of electric field at only the edge of the NTs’ rim (Additional file 1: Fig. S5a, b). These simulations suggested that even the 5 V pulses can generate sufficient electric field strength through the Au-coated NTs to surpass the electrical potential threshold of the cell membrane and allow for transient/reversible pore formation at the cell membrane of mammalian cells [114, 116–118]. An extended description of the electric field simulation is presented in Supplementary Sect. 2 (Additional file 1).

### Influence of ENI platform’s applied voltages on cell viability

To study the effects of the nanoscale-EP process on cell viability through the ENI platform, we applied two voltage amplitudes (5 and 10 V) in pulse form. A low-voltage function generator (0–10 V amplitude range) was used to apply a series of monophasic square-wave pulses to the ENI platform with defined electrical parameters: frequency 20 Hz, pulse-width 400 µs, number of cycles 600, and rise/fall time 5 ns.

To determine cell viability post nanoscale-EP, GPE-86 cells were stained with Hoechst 33,342 (Hoechst), propidium iodide (PI), and fluorescein diacetate (FDA)—indicating the nucleus, dead, and live cells, respectively. The confocal laser microscopy images and their quantification (Fig. 2b, c, respectively) showed the cell viability of 94.5 ± 1.5% at 5 V pulses, similar to that obtained for non-electroporated cells (0 V, 94.5 ± 2.5%) and was only slightly lower (90.1 ± 0.9%) at 10 V pulses (i.e., the most extreme condition considered). This indicated that the ENI platform causes minimal cell damage during the nanoscale-EP, with most cells recovering post nanoscale-EP. To avoid further reduction in cell viability post nanoscale-EP, we considered the working parameters 10 V, 400 µs, 20 Hz, 600 cycles to be the threshold for our ENI platform.

We also examined the influence of the applied voltages on the NTs’ structural integrity post nanoscale-EP by applying a series of 10 V pulses (with 400 µs pulse-width for 600 cycles) for three consecutive times. According to the SEM images of NTs before and after the nanoscale-EP (Additional file 1: Fig. S6a, b, respectively), the NTs’ Ti/Au coating remained intact, confirming the platform’s robustness post nanoscale-EP. Preservation of the NTs and their metal coating post nanoscale-EP allowed for samples to be easily cleaned, sterilized, and reused. In addition to reusability, the inherent rigidity of our Ti/Au limits the introduction of contaminations into the cell culture post nanoinjection.

### Diverse and tunable nanoinjection of targeted cargos into GPE-86 cells

After investigating cell viability post nanoscale-EP, we examined the performance and versatility of the ENI platform by delivering a range of bioactive cargos into the GPE-86 fibroblast cells, which have been reported to be relatively hard to transfect using conventional non-viral methods [119–121]. Four distinct membrane-impermeable biomolecules were selected: antibody, mRNA, pDNA, and siRNA. The remainder of this article will outline these investigations.

***Nanoinjection of antibodies****–* Delivery of antibodies into cells can have significant advantages in biomedical research that relies on internalization of antibodies for diagnostics, visualizations, and activity blocking; yet most antibodies demonstrate no cell-penetrating ability. So developing approaches for the efficient and safe delivery of antibodies and their derivatives into living cells is urgently needed for in situ detection and real-time monitoring [122–124].

**Fig. 3:**
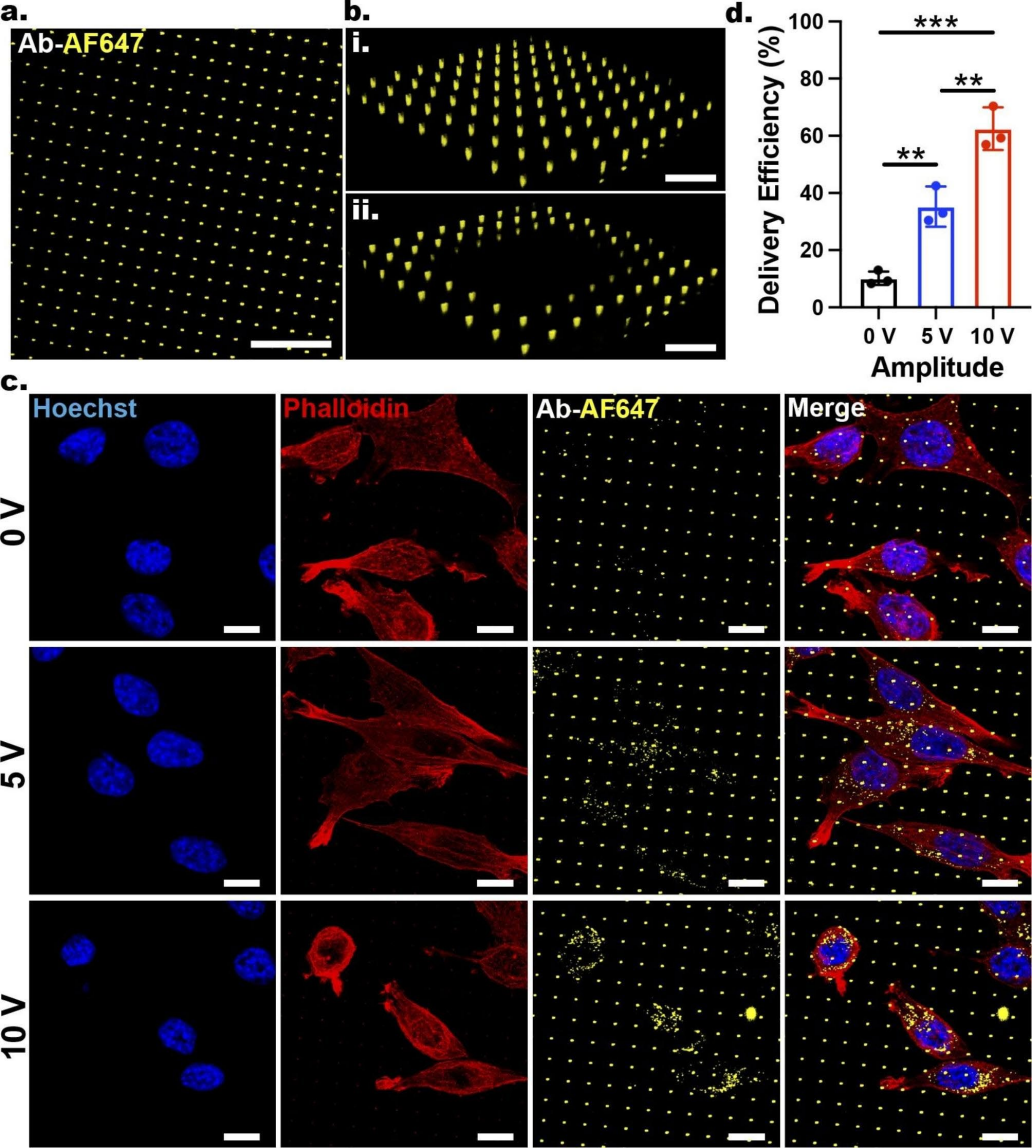
Nanoinjection of IgG­AF647 into GPE-86 cells via the ENI platform. **a** Loading of the AF647-tagged antibodies into the Au-coated NTs. Scale bar: 20 μm. **b** Comparison of the confocal microscopy images before (i) and after (ii) an intentional induced photobleaching. Scale bars: 10 μm. **c** Confocal microscopy images demonstrating the insertion and accumulation of IgG­AF647 into GPE-86 cells after the nanoscale-EP using two voltage amplitudes (5, 10 V) and the comparison to the non-electroporated cells (0 V). Scale bars: 10 μm. **d** Quantification of the percentage of cells containing IgG­AF647. Error bars indicate ± SDs, n = 3, **P ≤ 0.005, ***P = 0.0001, one-way ANOVA.

To explore the ENI platform’s potential to deliver antibodies into GPE-86 fibroblast cells, we first loaded Alexa Fluor 647-tagged goat anti-mouse IgG (IgG­AF647) into the NT arrays. Confocal microscopy images showed bright fluorescent spots at each NT location (Fig. 3a), indicating successful antibody loading within the NTs’ central cavity. To verify that the signal originated from the true cargo loading, we induced an intentional quenching step by increasing the laser intensity to its maximum level, causing photobleaching of the fluorescently tagged antibodies in a defined small region within the sample (Fig. 3b, i: before quenching and ii: after quenching).

Immediately after IgG­AF647 loading into the NT arrays, GPE-86 cells were cultured onto the NT arrays. After 3 h incubation we performed the nanoscale-EP at two working conditions (5 and 10 V; both with 20 Hz, 400 µs, 600 cycles). Cells cultured on IgG-loaded NTs but without an applied voltage served as the non-electroporated cells (0 V).

After a 30-min incubation period post nanoscale-EP, the cells were fixed with 4% paraformaldehyde (PFA) and stained with phalloidin­AF568 (phalloidin) and Hoechst. After 9 h incubation at 4 ºC, the cells were imaged via a laser confocal microscope.

Confocal microscopy images and their quantification (Fig. 3c, d) indicated that the ENI platform successfully delivered the fluorescently tagged antibodies with efficacy of 62.5 ± 7.4% (for 10 V) and 35.2 ± 7.0% (for 5 V), exhibiting IgG­AF647 insertion and accumulation within the cell. The accumulation of fluorescently tagged antibodies inside the cells in the obtained confocal microscopy images could be potentially due to the presence of aggregates of the injected cargos, leading to punctate signals, as opposed to a uniform distribution across the cytoplasm.

In contrast to 10 V treated cells, the non-electroporated cells (0 V) had 10.2 ± 2.6% delivery efficiency; this is likely to be due to the local cellular deformation and plasma membrane reorganization at the NTs that can facilitate the passive diffusion of IgG-AF647 into cells, as well as the NT-induced membrane curvatures that can recruit endocytic elements to enhance the cellular uptake of IgG-AF647 [15, 19, 35, 125].

The significant improvement of delivery efficiency obtained using 10 V pulses compared with 5 V pulses suggests that the use of higher voltage leads to stronger poration at the membrane–NT interface, facilitating more cargo uptake by the cells. The uptake of the cargos by the cells suggests that the cell membrane remained in close contact with the NTs during the nanoscale-EP. This was confirmed by cross-sectional view of NT–membrane interface before (0 V) and after the nanoscale-EP (5 and 10 V), which demonstrated that the plasma membrane remained intact post nanoscale-EP, maintaining a tight interface with the NTs (Additional file 1: Fig. S7). This can open opportunities for stable and long-term nanoscale-EP.

These results demonstrate the capability of the ENI platform to safely (evident by > 90% cell viability) and efficiently nanoinject proteins (62.5 ± 7.4%) into cells by applying precise electrical pulses with voltages that are significantly lower than the conventional BEP (from hundreds of Volts to ≤ 10 V). Lowering the applied voltage required for successful EP can significantly limit the undesired effects that can result in irreversible damage to cells post EP procedures, including the formation of reactive oxygen species, electrolytic reactions at the electrodes, and joule heating at the electrode surface—all of which stem from the excessive electric field during the EP experiments [1, 126, 127].

***Nanoinjection of mRNAs and pDNAs****–* Delivery of nucleic acids, DNAs and RNAs, into cells is a common approach for regulating cell function and behavior [128]. To demonstrate the applicability of the ENI platform to deliver nucleic acids into cells, we used Cy5-tagged mRNA and Cy3-tagged pDNA both encoding for green fluorescent protein (GFP). Preservation of the biological functionality of the delivered cargos was assessed by analyzing the level of GFP expression post nanoscale-EP using the ENI platform’s optimized parameters (10 V; 20 Hz; 400 µs; 600 cycles). Detection of Cy5/Cy3 signals were indicative of cargo internalization, while the GFP expression indicated the translational potency of the delivered mRNAs and pDNAs. To demonstrate the ability of our ENI platform to deliver diverse cargo types, regardless of their size, shape, and surface area, we selected the parameters that yielded the highest antibody nanoinjection (10 V; 20 Hz; 400 µs; 600 cycles) to successfully deliver mRNAs and pDNAs into cells.

Figure S8a, b (Additional file 1) show the confocal microscopy images for the successful loading of mRNAs and pDNAs, respectively, with bright fluorescent signals at each NT location. Similar to loading of the fluorescently tagged antibodies, we used the same intentional quenching step to verify the true florescence signals for both Cy5-tagged mRNAs (Additional file 1: Fig. S8a) and Cy3-tagged pDNAs (Additional file 1: Fig. S8b).

#### Nanoinjection of mRNAs

For mRNA delivery into GPE-86 cells through the ENI platform, the cells were seeded onto the mRNA-loaded NTs and cultured for 3 h, followed by the nanoscale-EP using the optimized parameters (10 V; 20 Hz; 400 µs; 600 cycles). Cells cultured on mRNA-loaded NTs but without an applied voltage served as the non-electroporated cells (0 V).

**Fig. 4:**
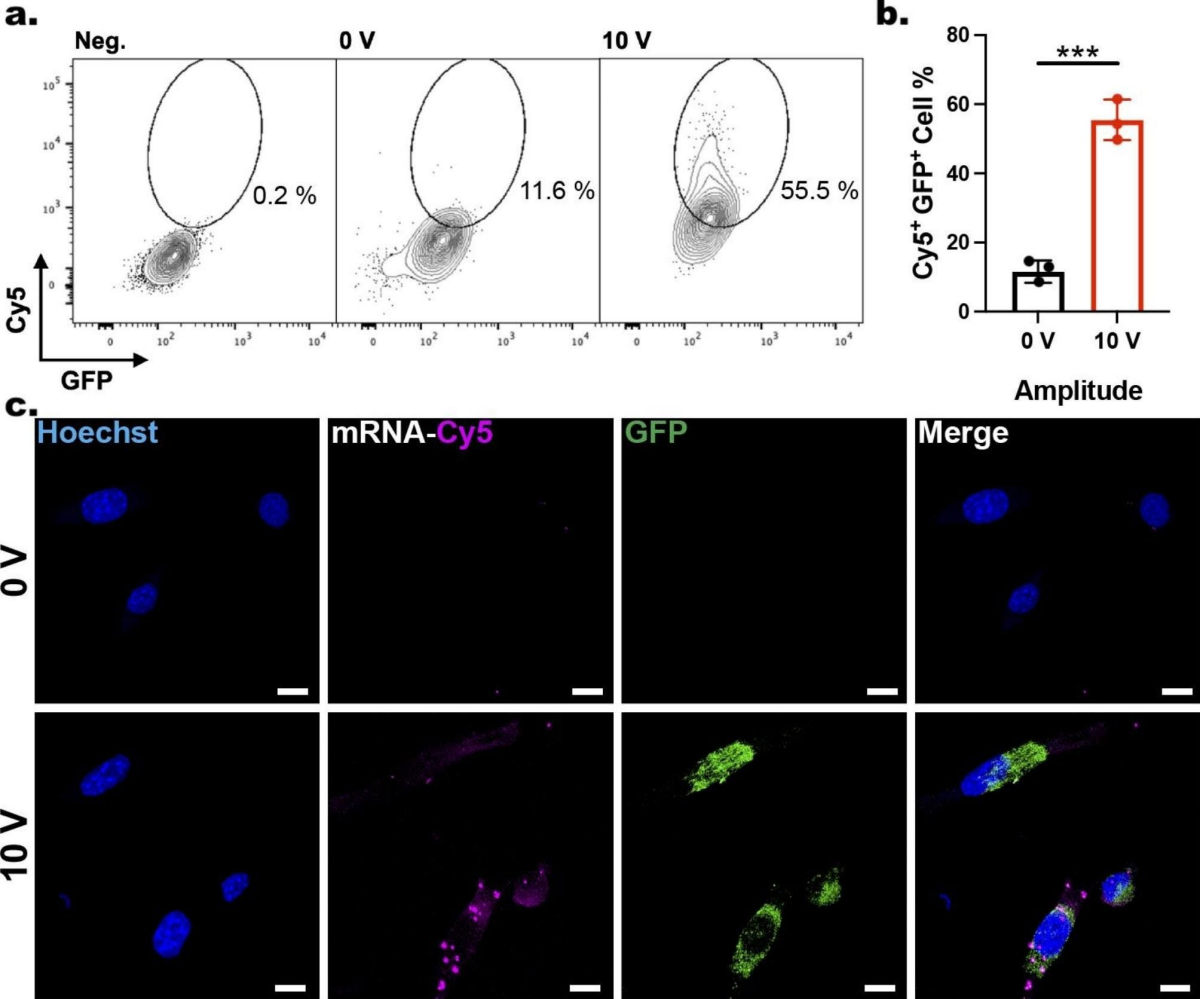
Nanoinjection of Cy5-mRNA-GFP into GPE-86 cells. **a** Flow cytometry analysis of GPE-86 cells (Negative control (Neg.), 0 V, and 10 V) detached from NTs pre-loaded with Cy5-mRNA-GFP with circle gating indicating Cy5^+^GFP^+^ population. **b** Quantification of the percentage of cells exhibiting both Cy5 and GFP signals. Error bars indicate ± SDs, n = 3, ***P = 0.0003, one-way ANOVA. **c** Confocal microscopy images demonstrating the insertion of Cy5-mRNAs into GPE-86 cells and the subsequent GFP expression for 0 and 10 V after 6 h. Scale bars: 10 μm

After the nanoscale-EP, the cells were harvested from the ENI platform using 0.25% trypsin-EDTA and cultured back in fresh media for a further 6 h. The cells were then processed and analyzed by flow cytometry (Fig. 4a). The quantification of Cy5^+^GFP^+^ population (Fig. 4b) showed that 55.5 ± 5.9% of the electroporated cells (10 V) contained Cy5 and exhibited GFP signal, suggesting successful insertion and translation of mRNAs without affecting their biological functionality. Quantification of non-electroporated cells (0 V) indicated an 11.6 ± 3.2% delivery efficiency—significantly lower than the electroporated cells. The cargo uptake by the non-electroporated cells (0 V) could likely be due to the passive diffusion of Cy5-tagged mRNAs from the NTs into the cells through enhanced endocytic pathways, caused by the tight cell membrane–NT interfacing and membrane deformation. To visualize the Cy5-mRNA-GFP delivery into the GPE-86 cells treated by the ENI platform, after a 30-min incubation period post nanoscale-EP, the cells were harvested and incubated in fresh culture media for 6 h. Immediately after, the cells were fixed with 4% PFA and stained with Hoechst. After 9 h incubation at 4 ºC, the cells were imaged via a laser confocal microscope. For the electroporated cells (10 V), a strong Cy5 accumulation was observed inside the GPE-86 cells with detectable GFP expression (Fig. 4c), indicating mRNAs internalization and their translation. By contrast, the non-electroporated cells (0 V) showed hardly any detectable Cy5 or GFP signals.

To assess the performance of the ENI platform against an existing state-of-the-art method, we compared the nanoinjection of mRNA efficacy with the popular BEP approach. In this comparison, cells were electroporated using a commercial electroporator (Bio-Rad Gene Pulser Xcell, Bio-Rad Laboratories). For this BEP approach investigation, we first used the mRNA quantity equivalent to the loading capacity of the NTs within the ENI platform for the chosen cell density (70 µL of 0.1 × 10^6^ cells/mL) for cell seeding, which was estimated to be 1.8 ng mRNA/10^6^ cells (direct comparison). The flow cytometry results showed delivery efficacy of 3.9 ± 0.3% for BEP (Additional file 1: Fig. S9a, b), indicating that the ENI platform (55.5 ± 5.9%) can produce a significantly higher transfection efficiency (14-fold higher) to that obtained via BEP (direct comparison), with much lower applied voltage (10 V for ENI platform compared with 200 V for BEP).

We also compared the performance of the ENI platform against BEP with the optimized concentration of mRNA that was provided by the manufacturer’s guideline (1.6 µg of mRNA/10^6^ cells; optimized); this is approximately 1000-fold higher mRNA quantity compared to the ENI approach, which can be attributed to the ability to exploit the NTs’ central cavity as reservoir for loading the targeted cargos. The results shown in Fig. S9a, b (Additional file 1) indicated a 50.9 ± 1.4% transfection efficiency for BEP with optimized condition; this was still lower than the transfection efficiency obtained via the ENI platform (55.5 ± 5.9%), despite requiring 1000-fold higher mRNA quantity and 20-fold higher voltage than the ENI platform. This further confirms the ENI platform’s improved performance over conventional BEP.

**Fig. 5:**
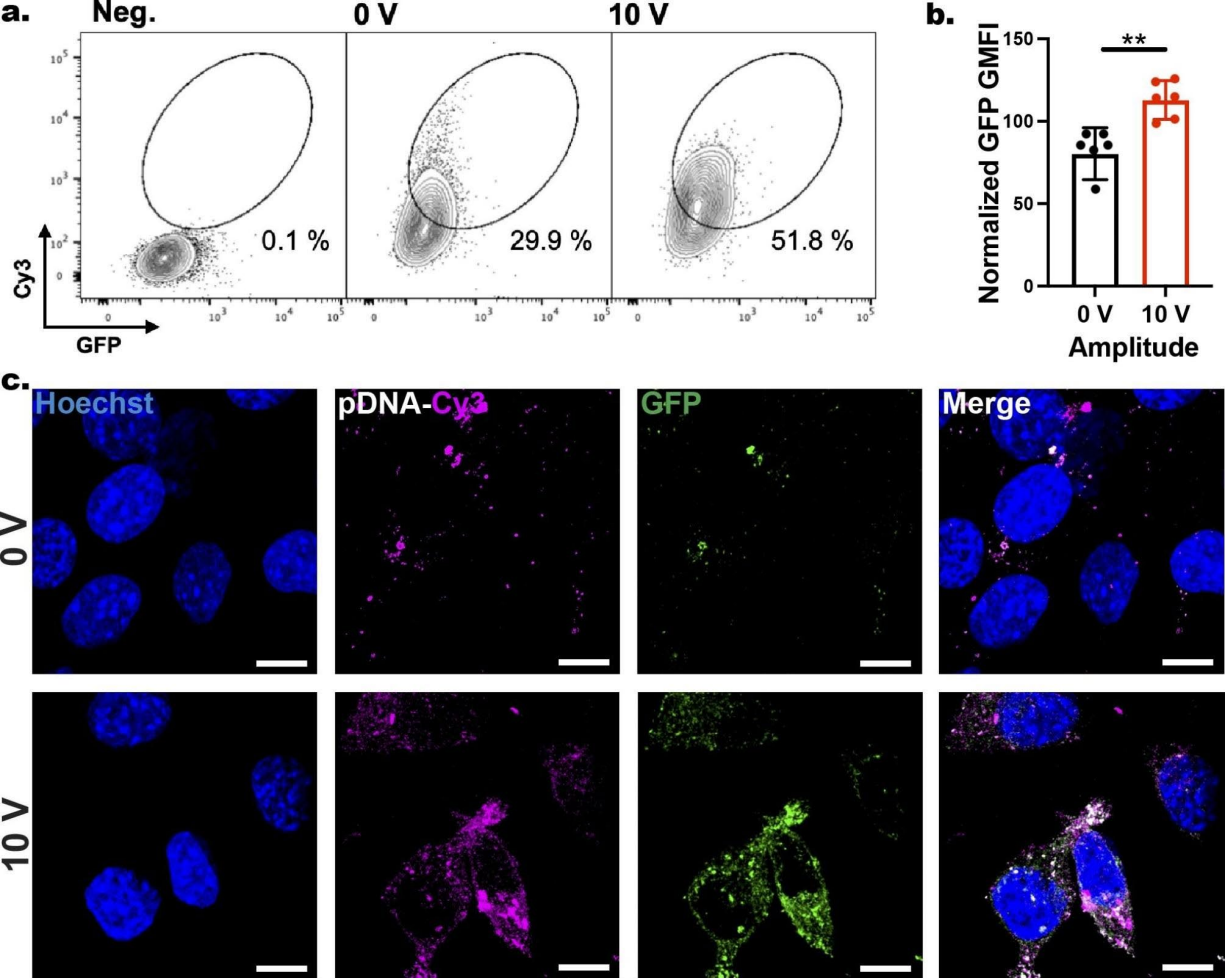
Nanoinjection of Cy3-pDNA-GFP into GPE-86 cells using the ENI platform. **a** Flow cytometry analysis of GPE-86 cells (Negative control (Neg.), 0 V, and 10 V) detached from NTs that were pre-loaded with Cy3-pDNA-GFP. Circle gating indicating Cy3^+^GFP^+^ population. **b** Plot showing geometric mean fluorescence intensity (GMFI) of GFP expression in cells from non-electroporated (0 V) and electroporated (10 V) samples. Error bars indicate ± SDs, n = 6, **P = 0.002, unpaired t-test. **c** Confocal microscopy images demonstrating the Cy3 signal in GPE-86 cells and their GFP expression for 0 and 10 V after 24 h. Scale bars: 10 μm

*Nanoinjection of pDNAs*: We next tested the delivery of fluorescently tagged pDNAs encoded for GFP into GPE-86 cells using the ENI platform (Fig. 5; Cy3-tagged pDNAs) and in primary human T cells (Cy5-tagged pDNAs; Additional file 1: Supplementary Sect. 1.5 and 1.6; Fig. S10). After the nanoscale-EP, the GPE-86 cells were harvested from the ENI platform using 0.25% trypsin-EDTA and cultured back in fresh media for a further 24 h. The results indicated an increase in Cy3^+^GFP^+^ population from 29.9% in non-electroporated (0 V) to 51.8% in electroporated (10 V) samples (Fig. 5a). The Cy3^+^GFP^+^ signal observed in the non-electroporated cells (0 V) could be explained by the passive diffusion of Cy3-tagged pDNAs from the NTs into the cells through endocytic pathways, caused by localized stress due to deformation of the membrane by the NTs [1, 35, 40]. Yet, despite relatively high level of Cy3 signal in the non-electroporated cells (0 V), it was evident that the GFP intensity was significantly higher (by 40%) in the electroporated cells (10 V) than that of the non-electroporated cells after 24 h (Fig. 5b). The higher GFP expression in electroporated cells (10 V) compared with non-electroporated cells (0 V) is likely to be a consequence of enhanced influx of the pDNAs into the cells through the transient pores during nanoscale-EP, augmenting the effect of plasma membrane remodeling that is responsible for stimulating endocytic pathways in non-electroporated cells [1, 15].

To visualize the Cy3-pDNA-GFP delivery into the GPE-86 cells treated by the ENI platform, after a 30-min incubation period post nanoscale-EP, the cells were harvested and incubated in fresh culture media for 24 h. Immediately after, the cells were fixed with 4% PFA and stained with Hoechst. After 9 h incubation at 4 ºC, the cells were imaged via a laser confocal microscope. The results from confocal microscopy were in line with the flow cytometry data, showing Cy3^+^GFP^+^ cells for both 0 and 10 V samples; but stronger Cy3 and GFP signals were observed for the electroporated cells (Fig. 5c).

### Gene silencing via nanoinjection of siRNAs

To demonstrate the potential use of the ENI platform in advanced cellular manipulation, we delivered siRNA—an effective tool to knockdown gene expression—into GPE-86 cells. The selected siRNA was customized to specifically target TRIOBP, an F­actin bundling protein that is crucial for actin skeleton reorganization, cell migration, and proliferation [129, 130]. Successful delivery of anti-TRIOBP siRNAs would result in knockdown of TRIOBP expression level in GPE-86 cells, which can severely impact actin meshwork and filopodial formation, and therefore altering the cellular morphology [131].

For this investigation, GPE-86 cells were cultured on the NTs loaded with anti-TRIOBP siRNAs for 3 h, and proceeded without (0 V) or with nanoscale-EP (10 V; 20 Hz; 400 µs; 600 cycles) using the ENI platform. Cells electroporated via the ENI platform with scramble siRNAs (Neg. siRNAs) loaded into the NTs served as the negative control, as the scramble siRNAs should have insignificant impact on cellular morphology when inserted into the cells.

**Fig. 6:**
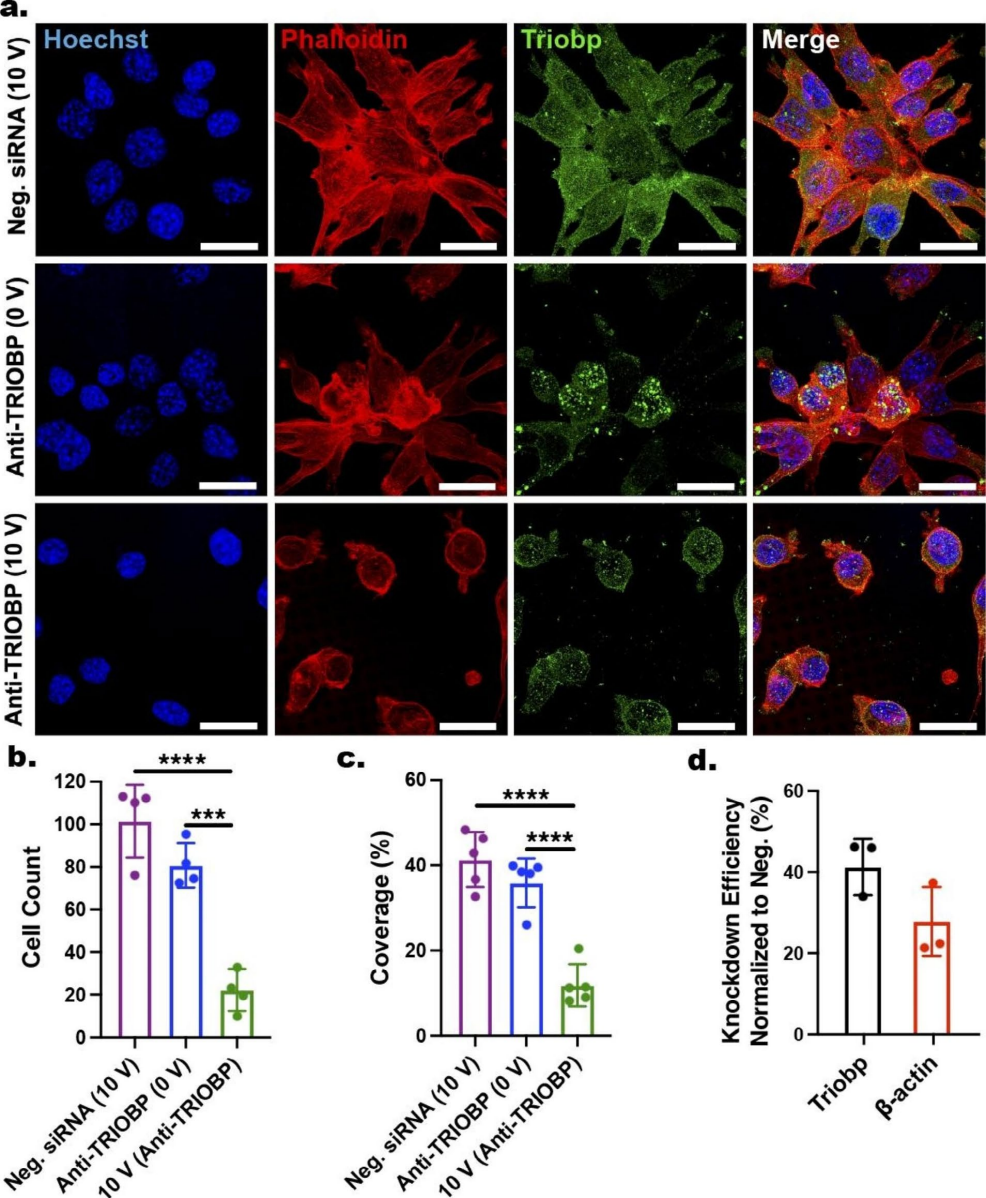
Nanoinjection of siRNAs for targeted gene knockdown. **a** Confocal microscopy images showing GPE-86 cells after 24 h culture on Au-coated NTs before and after the nanoscale-EP (10 V; 400 µs; 20 Hz; 600 cycles) that were pre-loaded with scramble (Neg. siRNA, 10 V) or TRIOBP-targeting siRNAs at two working conditions (0 and 10 V). Cells were stained with Hoechst (blue), phalloidin (red), and TRIOBP antibody (green). Scale bars: 20 μm. **b** Comparison between the three conditions in terms of number of cells within defined regions. Error bars indicate ± SDs, n = 4, ***P = 0.0003 and ****P < 0.0001, one-way ANOVA. **c** Comparison between the three conditions in terms of cell coverage. Error bars indicate ± SDs; n = 5, ****P < 0.0001, one-way ANOVA. **d** Knockdown efficiency of TRIOBP and β-actin in electroporated cells (10 V) on NTs loaded with anti­TRIOBP siRNA. Error bars indicate ± SDs, n = 3, unpaired t-test

After 24 h, the cells were fixed and stained with Hoechst, phalloidin, and TRIOBP antibody and imaged via confocal laser microscopy for detailed visualization of cellular morphology, TRIOBP expression and localization, and actin cytoskeleton organization of the GPE-86 cells on the NT arrays (Fig. 6a). According to the confocal microscopy images, the electroporated cells (10 V) that were cultured on the NTs (with anti­TRIOBP siRNA loading) exhibited significantly lower expression of TRIOBP, evident by the drastic change in cellular morphology and their inability to spread on the NT arrays. By contrast, both the non-electroporated cells cultured on the NTs loaded with anti­TRIOBP siRNAs (0 V) and the electroporated cells cultured on NTs loaded with scramble siRNA (Neg. siRNA (10 V)) showed negligible changes in their cellular morphology. Additionally, large­scan confocal microscopy images (Additional file 1: Fig. S11) and their quantitative analysis via ImageJ (Fig. 6b, c) demonstrated higher amount, larger coverage, and more even distribution of GPE-86 cells on NT arrays for the anti­TRIOBP (0 V) and scramble siRNA (10 V) groups, compared with that for the anti­TRIOBP group (10 V). These results confirm the successful delivery of anti­TRIOBP siRNAs into GPE-86 cells via the ENI, where the delivered anti­TRIOBP siRNAs performed their functionality of specifically targeting TRIOBP expression, resulting in impaired support for actin cytoskeleton, filopodial formation, and thus focal adhesion of GPE86 cells on the NTs [130].

To further quantify gene knockdown efficiency of ENI-delivered siRNAs, we first lysed the cells and extracted total RNAs after the nanoscale-EP (10 V) with anti­TRIOBP or Neg. siRNAs, and then performed a real-time quantitative reverse transcription - polymerase chain reaction (qRT-PCR) analysis. The expression of TRIOBP and β-actin was measured and normalized to that of a housekeeping gene (glyceraldehyde 3-phosphate dehydrogenase, GAPDH). The qRT-PCR results confirmed significantly reduced expression (at mRNA transcriptional level) of TRIOBP in electroporated cells treated with anti­TRIOBP siRNAs, with gene knockdown efficiency quantified as 41.3 ± 6.9% (Fig. 6d). Interestingly, we also observed a reduced level of β-actin expression (27.9 ± 8.5%) in cells treated with anti­TRIOBP siRNA via ENI platform, suggesting indirect impact of knocking down TRIOBP on actin expression, and consequently reorganization of actin cytoskeleton in cells [131].

## Conclusion

In this study, we developed a non-viral, low-voltage, and reusable ENI platform, and showed its applicability to efficiently deliver a diverse range of cargos into Mouse embryonic fibroblast cells (GPE-86). We demonstrated the ability to create highly ordered vertically aligned NTs with precise control over geometrical parameters. This fabrication route also allowed the samples to be cleaned and reused. This was evident by the SEM images obtained of the NTs after exposing the samples to the chosen electric pulses (10 V; 20 Hz; 400 µs; 600 cycles) that did not show any damage to the structural integrity and the conductive coating of the NTs, even after three consecutive times post nanoscale-EP. This could be attributed to two factors: the high stability of the Ti layer underneath the Au layer and the use of ultra-short and low-voltage and ultra-short pulses (10 V at 400 µs). This reusability can potentially reduce the cost of operation. Exploiting the NTs’ inner cavity as reservoirs enabled direct cargo loading and eliminated the need for integrating microfluidic channel underneath. The programmable NTs’ topography prompted tight adhesion of the cellular membrane to NTs, allowing for localization of the electric fields at the NT–membrane interface; this significantly lowered the applied voltage to form transient pores across the cell membrane during the nanoscale-EP, allowing most cells to recover post nanoscale-EP. In the context of mRNA delivery, we conducted a comparison between the ENI platform and BEP. The results demonstrated that the ENI platform achieved higher transfection efficiency despite requiring 1000-fold lower mRNA quantity and 20-fold lower voltage. This comparison confirms the superior performance of the ENI platform over conventional BEP. In addition to successful delivery of a broad range of exogenous materials regardless of their type, size, and surface charge into fibroblast cells (i.e., proteins and nucleic acids), we also showed that the ENI platform can be used to efficiently induce gene knockdown by delivering siRNAs into fibroblast cells. Such ENI platform holds great potential for delivering advanced therapeutics, with minimal cellular perturbation, opening new pathways for advanced ex vivo cellular engineering. Additionally, in context of large-scale fabrication of this intracellular delivery platform, the nanoimprint lithography can be a promising candidate; this is due to its cost-effectiveness and high throughput [132–134]. For the large-scale production of this NT-assisted EP platform, the nanoimprint lithography could be adapted to imprint nanoscale patterns into a substrate, followed by the deposition and etching processes to create the nanotubes. The process could be optimized to control the NT dimensions and spacing to meet the specifications for EP procedures.

## Experimental section

### Fabrication of Au-coated vertically aligned Si nanotubes (VA-SiNTs)

The detailed fabrication process is described in Supplementary Sect. 1.1 (Additional file 1). In brief, a flat Si wafer (4”, p-type, 3–6 Ωcm, < 100>; Siltronix) was first coated with a negative resist (6% hydrogen silsesquioxane (HSQ) – XR-1541-006; Dow Corning) at 1500 rpm for 1 min. Using an electron beam lithography (EBL) system (VISTEC EBPG-5000+; Raith Company) the desired patterns were formed with an accelerating voltage of 100 kV, beam current of 30 nA, and a dose of 1400 µCcm^− 2^. The exposed pattern consisted of arrays of ring structures (120–160 nm in height) with 300 nm and 500 nm for inner- and outer-diameter, respectively. Next, the unexposed resist was removed by immersing the wafer in the AZ726MIF developer for 30 s. The resist ring structures were then used as masks during the top-down dry etching process using the ULVAC NLD5700 DRIE with simultaneous flow of SF_6_ and O_2_ at a pressure of 1 Pa. The Antenna RF power and Bias RF/LF power were set to 200 W and 16 W, respectively. Helium pressure was set at 2000 Pa and the circulator at − 20 ºC. The etching time was set to 145 s to produce 2 µm tall NTs. Post etching, the wafer was cut into 1 cm × 1 cm pieces and cleaned with piranha (3:1, H_2_SO_4_:H_2_O_2_ v/v) for 10 min. Next, at 10^− 7^ Torr, a thin layer of Ti (10 nm) and Au (50 nm) was deposited onto the samples using a DC/RF sputtering system (Hummer BC-20; Anatech).

### Fabrication of biocompatible enclosure

The biocompatible enclosure was fabricated using polydimethylsiloxane (PDMS; Sylgard 184, Dow Corning, US). In this process, first the silicon base and the curing agent components were mixed at a 1:10 ratio. Immediately after, 0.8 mL of the PDMS mixture was poured into a 24-well plate. After 2 h in the oven (70 ºC), the cured PDMS pieces were cut into 8 mm × 8 mm pieces. To form an opening consistent with the size of the pattern area (3 mm × 3 mm), a 5 mm micro-punch was used.

### Construction of holder for the ENI platform

The custom holder was constructed using a 3D resin printer (J826; Stratasys) with a biocompatible resin (VeroContactClear; Tri-Tech 3D Ltd) to house three samples (1 cm × 1 cm pieces, each with 3 mm × 3 mm pattern area) at once with separate wells for each sample (Additional file 1: Supplementary Sect. 1.2).

### Cell culture

The mouse embryonic fibroblast cells (GPE-86; ATCC, CRL-9642) cells were grown and maintained in complete Dulbecco’s modified Eagle’s medium (DMEM, Gibco) supplemented with 10% fetal bovine serum (FBS, Gibco), 1 mM sodium pyruvate (Gibco), 2 mM L-glutamine (Gibco), 100 U mL^–1^ penicillin (Gibco), and 100 µg mL^–1^ streptomycin (Gibco). Cells were incubated at 37 ˚C with 5% CO_2_.

### Nanoscale-EP process via the ENI platform

To perform nanoscale-EP using the ENI platform, a Function/Arbitrary Waveform Generator (33,220 A; Agilent Technologies) was used. After incubating the cells onto the PDL-coated NTs for 3 h at 37 ºC and 5% CO_2_, the samples were placed in the custom holder and series of monophasic square-wave pulses were applied to each sample separately (amplitudes 5 and 10 V; frequency 20 Hz; pulse width 400 µs; number of cycles 600). Post nanoscale-EP, the samples were placed in fresh complete DMEM (Additional file 1: Supplementary Sect. 1.3).

### Conventional bulk EP (BEP)

For delivery of Cy5-mRNA-GFP into GPE-86 cells via conventional BEP, we used the Bio-rad Gene Pulser Xcell Electroporation System and Gene Pulser electroporation buffer (Bio-Rad Laboratories, US) (Additional file 1: Supplementary Sect. 1.4).

### Cell viability assay

The cell viability after the nanoscale-EP was assayed by live–dead staining using fluorescein diacetate (FDA; Sigma-Aldrich) and propidium iodide (PI; Sigma-Aldrich). FDA produces green fluorescence in live cells (excitation 498 nm, emission 517 nm), while PI only enters the cells with damaged membrane and produces red fluorescence in dead cells (excitation 535 nm, emission 617 nm). Using a final concentration of 15 µg/mL for FDA and 10 µg/mL for PI, the samples were incubated in complete DMEM at 37 ºC for 5 and 15 min, respectively. In addition to live–dead dyes, Hoechst 33,342 (Hoechst; Sigma-Aldrich) was added to the cells at a final concentration of 1 µg/mL and incubated for 15 min at 37 ºC to stain the nucleus, helping to locate and identify the total number of cells (excitation 340 nm, emission 510 nm). After the incubation period, the samples were rinsed three times with DPBS before being observed though the confocal laser scanning microscope system (Stellaris 5; Leica). All experiments were repeated at least three times.

### Confocal laser scanning microscopy imaging

To visualize the cells, Leica Stellaris 5 confocal laser scanning microscopy system (inverted) was used. Each sample was imaged at five different regions (i.e., middle and four corners). For viability, 20× magnification non-immersive objective lens was used. For more detailed imaging, the magnification of 60× oil-immersive objective lens was used. To obtain super high-resolution images via the Leica Stellaris 5 confocal laser scanning microscopy system, the lightning mode was used. Images were analysed using Leica Application Suite X provided by the manufacturer and ImageJ.

### Cell fixation for confocal laser scanning microscopy imaging

Cells were washed with DPBS and then fixed in a solution of 4% paraformaldehyde (PFA; Electron Microscopy Sciences) for 10 min at room temperature in dark. After fixation, the cells were washed twice with DPBS. The cells were then stained with Alexa Fluor 568 Phalloidin (Phalloidin; Thermo Fisher) and Hoechst to stain actin filaments (F-actin) and nucleus, respectively. For cells treated with scramble and anti-TRIOBP siRNAs, after PFA fixation, the cells were permeabilized using 0.1% Triton X-100 (Sigma-Aldrich) in DPBS for 5 min at RT for the subsequent staining.

### Cell fixation for SEM and FIB-SEM

Cells grown on NT arrays were rinsed with 0.1 M sodium cacodylate buffer (Electron Microscopy Sciences) and fixed with 2.5% glutaraldehyde (Electron Microscopy Sciences) in 0.1 M sodium cacodylate at 4 ºC overnight. Following this, samples were washed (3 × 5 min) with chilled 0.1 M sodium cacodylate buffer and post-fixed with 1% osmium tetroxide (Electron Microscopy Sciences) in 0.1 M sodium cacodylate at room temperature for 1 h. After repeating the washing step, samples were gradually dehydrated with increasing concentrations of ethanol; 50%, 70%, 90% (1 × 10 min) and 100% (2 × 10 min) at room temperature, and finally critical point dried (CPD 030 Critical Point Dryer, BAL-TEC). Samples were then mounted on SEM stubs and sputter-coated with a thin layer (~ 7 nm) of either Au or Pt to increase their conductivity.

### Staining of intracellular compartments for FIB-SEM

The sample preparation combined heavy metal staining with resin embedding. Samples were rinsed with 0.1 M sodium cacodylate buffer and fixed with 2.5% glutaraldehyde in the same buffer at 4 ºC overnight. Following this, samples were washed (3 × 5 min) with chilled 0.1 M sodium cacodylate buffer and quenched with chilled 20 mM glycine solution (Sigma-Aldrich) in the same buffer for 20 min. After repeating the washing step, samples were post-fixed by combining equal volumes of 4% aqueous osmium tetroxide with 2% potassium ferrocyanide (UNIVAR) in 0.2 M sodium cacodylate buffer on ice for 1 h. Samples were then washed again (3 × 5 min) with chilled buffer and incubated with 1% tannic acid (BDH) in water at room temperature for 20 min. After rinsing with buffer (2 × 5 min) samples were further incubated with 2% aqueous osmium tetroxide at room temperature for 30 min. Following this, samples were washed (2 × 5 min) with distilled water and incubated with syringe-filtered 4% aqueous uranyl acetate (UNIVAR) at 4 ºC overnight. Samples were then washed (3 × 5 min) with chilled distilled water and gradually dehydrated with increasing concentrations of ethanol; 10%, 30%, 50%, 70%, 90% and 100% (1 × 7 min) at room temperature. An epon resin 20 mL solution was prepared by initially mixing 12.2 g of DDSA (Dodecenyl Succinic Anhydride Specially Distilled 13,710, Electron Microscopy Sciences), 4.4 g of Araldite (GY 502 10,900, Electron Microscopy Sciences) and 6.2 g of Procure 812 (EMBED 812 RESIN 14,900) using a mechanical stirrer. Once the solution was uniformly mixed, 0.8 mL of BDMA (N-benzyldimethylamine 11,400, Electron Microscopy Sciences) was added to it while stirring. Samples were then infiltrated with increasing concentrations of the freshly prepared resin solution in 100% ethanol at room temperature and in a sealed container using the following ratios: 1:3 (3 h), 1:2 (3 h), 1:1 (overnight), 2:1 (3 h), 3:1 (3 h). Following this, samples were finally infiltrated with 100% resin solution overnight. Prior to polymerization at 60 ºC, the excess resin was drained away by mounting the samples vertically for 1 h.

### SEM imaging

SEM imaging of the samples was performed on a Nova NanoSEM 430 (FEI; Thermo Fisher). The images were taken at tilt (45º) or top views with an electron beam acceleration voltage of 3–5 kV and a current of 28 and 80 pA, while using a secondary electron detector.

### FIB sectioning and imaging

FIB sectioning of the samples was performed using a Helios G4 UX FIB-SEM (Thermo Fischer) vertically and at 45 ° to the sample surface. Prior to FIB sectioning, the region of interest was protected from ion beam (i-beam) damage using i-beam assisted deposition of a ~ 0.5 μm thick Pt layer. The coating was carried out at 30 kV using i-beam current of 0.26–0.44 nA, depending on the area size. Following this, rough milling was performed at acceleration voltage of 30 kV and a current of 20 nA. The resulting cross sections were then polished with a voltage of 30 kV and a current ranging between 1.2 and 2.4 nA. Images were taken using an electron beam at acceleration voltage of 3 kV and a current of 200 pA using immersion mode and with a TLD detector operating in backscattered electron collection mode, at a dwell time of 5 µs and 6144 × 4096 pixel resolution. During sequential sectioning, images were taken every 20 nm using previously mentioned e-beam conditions.

#### Flow cytometry

An LSR Fortessa X20 flow cytometer (BD) was used to investigate the transfection efficiency of cells harvested from the NT arrays. Before analysis, cells were centrifuged in v-bottom 96-well plate and washed twice with flow cytometry staining buffer (FACS buffer). Cells were stained with Zombie Aqua™ Fixable Viability Kit (BioLegend) as per manufacturer’s instruction before flow cytometry analysing.

### Real-time quantitative reverse transcription-polymerase chain reaction (qRT-PCR)

Total RNA was isolated from GPE-86 cells 24 h after siRNA (Neg. or anti-TRIOBP) transfection by ENI using the RNeasy Mini Kit (Qiagen). Total RNA was reverse transcribed using Kit (Thermo Fisher) with random primers as per manufacturer’s instruction. Complementary DNA (cDNA) was then quantified using the Accupower Q-PCR kit (Bioneer) with the following primers: TRIOBP forward 5´-GACGAAGACGAGGACCCTAA-3´ and reverse 5´-ATCATCTCTGTGAGTCTGTTCC-3´; GAPDH forward 5´-ACCACAGTCCATGCCATCAC-3´ and reverse 5´- TCCACCACCCTGTTGCTGTA-3´; β-actin forward 5´ CCAACCGTGAAAAGATGACC-3´ and reverse 5´- GAGGTAGTCTGTCAGGTCCC-3´.

#### Data processing and statistical analysis

Fluorescence and SEM images were processed and analyzed by Image J. False coloring of SEM images was done using Photoshop (Adobe). Flow cytometry data were analyzed with FlowJo. All statistical analysis was performed using Prism GraphPad 9. Unpaired t-tests were performed for comparison between two groups. One-way ANOVA was used to calculate univariate data set with more than two groups. Quantification of the percentage of cells containing IgG-AF647 (delivery efficiency (%)) was done by considering the total number of cells with positive signal against the total number of cells in a set view; confocal images were obtained from different regions across the pattern area (3 mm × 3 mm).

#### Theoretical simulations of electric field across the ENI platform

The commercial software Ansys (2020 R1, Maxwell 2D module) was utilized to simulate and analyze the electric field distribution across the platform during the nanoscale-EP (Additional file 1: Supplementary Sect. 2).

### Electronic supplementary material

Below is the link to the electronic supplementary material.

Supplementary Material 1

Supplementary Material 2

## Data Availability

The data that support the findings of this study are available from the corresponding author upon reasonable request.

## List of abbreviations

ENI: Electroactive nanoinjection
EP: Electroporation
BEP: Bulk electroporation
NWs: Nanowires
NSs: Nanostraws
NTs: Nanotubes
VA-SiNTs: Vertically aligned silicon NTs
pDNA: Plasmid DNA
siRNA: Small interfering RNA
PDMS: Polydimethylsiloxane
PDL: Poly-D-lysine
PI: Propidium iodide
FDA: Fluorescein diacetate
GFP: Green fluorescent protein
qRT-PCR: Quantitative reverse transcription - polymerase chain reaction
GAPDH: glyceraldehyde 3-phosphate dehydrogenase
