# Supplementary material for: Electroactive nanoinjection platform for intracellular delivery and gene silencing
Source: J Nanobiotechnology. 2023 Aug 17;21:273. doi: 10.1186/s12951-023-02056-1 (PMC10433684; doi:10.1186/s12951-023-02056-1)
Supplement: Supplementary file 1 — Supplementary Material 1 [file 12951_2023_2056_MOESM1_ESM.docx]

Supplementary information

Electroactive nanoinjection platform for intracellular delivery and gene silencing

Ali-Reza Shokouhi^1,2,†^, Yaping Chen^1,2, †^, Hao Zhe Yoh^1,2^, Takahide Murayama^3^, Koukou Suu^3^, Yasuhiro Morikawa^3^, Jason Brenker^4^, Tuncay Alan^4^, Nicolas H. Voelcker^1,2,5,6,^*, and Roey Elnathan^1,2,7,8,9,^*

^1^ Monash Institute of Pharmaceutical Sciences, Monash University, 381 Royal Parade, Parkville, VIC 3052, Australia

^2^ Melbourne Centre for Nanofabrication, Victorian Node of the Australian National Fabrication Facility, 151 Wellington Road, Clayton, VIC 3168, Australia

^3^ Institute of Semiconductor and Electronics Technologies, ULVAC Inc., 1220-1 Suyama, Susono, Shizuoka, 410-1231, Japan

^4^ Department of Mechanical and Aerospace Engineering, Monash University, Wellington Rd, Clayton, VIC 3168, Australia

^5^ INM-Leibniz Institute for New Materials, Campus D2 2, Saarbrücken 66123, Germany

^6^ Department of Materials Science and Engineering Monash University, 22 Alliance Lane, Clayton, VIC 3168, Australia

^7^ Faculty of Health, School of Medicine, Deakin University, Waurn Ponds, VIC 3216, Australia

^8^ Institute for Frontier Materials, Deakin University, Geelong Waurn Ponds campus, VIC, 3216, Australia

^9^ The Institute for Mental and Physical Health and Clinical Translation, School of Medicine, Deakin University, Geelong Waurn Ponds Campus, Melbourne, VIC, 3216, Australia

*Corresponding author E-mail:

[nicolas.voelcker@monash.edu](mailto:nicolas.voelcker@monash.edu), [roey.elnathan@monash.edu](mailto:roey.elnathan@monash.edu)

†These authors contributed equally.

1. Supplementary text

- 1. **Fabrication of Au-coated vertically aligned Si nanotubes (VA-SiNTs):** In this fabrication process, a flat Si wafer (4", p-type, 3–6 Ωcm, <100>, Siltronix, France) was first cleaned with piranha (3:1, H_2_SO_4_:H_2_O_2_ v/v) for 1 h to remove organic contaminants from the surface. The wafer was then thoroughly rinsed with deionized (DI) water and dried under a nitrogen jet. Next, a negative resist (6% hydrogen silsesquioxane (HSQ) – XR-1541-006; Dow Corning, USA) was spin-coated onto the wafer at 1500 rpm for 1 min. The wafer was then directly loaded into an electron beam lithography (EBL) system (VISTEC EBPG-5000+; Raith Company, Germany). The desired patterns were then formed within the resist using EBL at an accelerating voltage of 100 kV with a beam current of 30 nA and a dose of 1400 μCcm^-2^. The exposed pattern consisted of arrays of ring structures (120–160 nm in height) with desired inner- and outer-diameters (300 and 500 nm, respectively). After the EBL exposure, the unexposed resist was removed by immersing the wafer in the AZ726MIF developer for 30 s. Development was then stopped with DI water and the wafer was thoroughly rinsed with DI water and dried under a nitrogen jet. The resist ring structures were then used as masks during the top-down dry etching process. Before etching, the Si wafer was cut into four 3 cm × 3 cm pieces. For the etching process, the samples were loaded into the ULVAC NLD5700 DRIE and etched in a simultaneous flow of SF_6_ and O_2_ at a pressure of 1 Pa with Antenna RF power of 200 W and Bias RF/LF power of 16 W. Helium pressure was set at 2000 Pa and the circulator at –20 ºC. The etching time was set to 145 s to produce 2 µm tall NTs. Post etching, the samples were cleaned with ethanol, acetone, and DI water, followed by drying under a nitrogen jet. Next, the 3 cm × 3 cm pieces were cut into 1 cm × 1 cm pieces and cleaned with piranha (3:1, H_2_SO_4_:H_2_O_2_ v/v) for 10 min. The sample pieces were then thoroughly rinsed with DI water and dried under a nitrogen jet. To form a conductive layer on the surface of the NTs, the sample pieces were loaded into a DC/RF sputtering system (Hummer BC-20 Anatech). At 10^-7^ Torr, first a thin layer of Ti (10 nm) was sputter coated onto the samples. Next, without breaking the vacuum, Au was deposited onto the samples (50 nm). The fabrication process is illustrated in Scheme S1.
  2. **Construction of holder for the ENI platform:** A custom holder was constructed using a 3D resin printer (J826, Stratasys, Isreal) with a biocompatible resin (VeroContactClear, Tri-Tech 3D Ltd, UK). To simplify the experimental process, the holder was designed to house and process three samples (1 cm × 1 cm pieces, each with 3 mm × 3 mm pattern area at the center)) at once with separate wells for each sample. As shown in Fig. S2, the holder consisted of two main pieces, held together using four magnets. The top portion of the holder comprised of three wells (height = 8 mm, width = 6 mm, length = 6 mm) for the electrolyte, and a hollow region (diameter = 2 mm) for housing the Pt rod-shaped electrode (eDAQ Pty Ltd, Australia). A 100 µm thick PDMS was attached underneath the top portion to avoid damaging the samples, as well as creating a tight seal to prevent leakage in the holder. The bottom portion of the holder consisted of a thin PDMS layer to hold the samples securely and prevent any damage to the samples from underneath. The constructed holder was designed to maintain a 200 µm gap between the samples and the Pt electrode once closed. To make electrical connections within the system, a Cu tape was placed on the edge of each sample. Once the holder is closed, the Cu tape is pressed onto the sample, forming a secure and robust electrical connection. The PDMS layer underneath the top portion of the holder was designed to shelter the Cu tape from the media during the nanoscale-EP process. Each well in the holder was designed to hold 150 µL of culture media.
  3. **ENI platform’s operation:** The complete process of the ENI platform’s operation is demonstrated in Scheme S2. In this process, first the ethanol and UV sterilized PDMS enclosure was placed on the PDL-coated sample. Next, the cargo solution (5 µL) was added to the PDMS enclosure, covering the pattern area and allowing the cargos to get into the NTs. After 1 h incubation on the orbital shaker (30 rpm), the excess cargo solution was removed using an auto pipette and the sample was rinsed with cell culture media to remove unloaded cargos. Next, the cell suspension was added to the PDMS enclosure (70 μL of 0.1 × 10^6^ cells/mL in cell culture media). After a 3 h incubation period at 37 ºC and 5% CO_2_, the PDMS enclosure was peeled-off and the sample was processed via nanoscale-EP by placing it in the 3D printed holder. Next, the sample was incubated at 37 ºC and 5% CO_2_ for 30 min in fresh cell culture media. To only analyze the cells on the pattern area, the cells outside of the pattern area were removed using a plastic scraper (i.e., physical means). For live cell imaging through fluorescent/laser confocal microscopy, the cells were stained without removing the cells from the pattern area. For detailed imaging, the cells were fixed using 4% paraformaldehyde (PFA; Electron Microscopy Sciences) for 10 min. For analysis through more efficient quantitative methods (e.g., flow cytometry), 0.25% trypsin was used to detach the cells from the platform. To perform real-time quantitative reverse transcription - polymerase chain reaction (qRT-PCR) analysis, the cells were first lysed on the platform and the RNAs were extracted.
     1. **Surface functionalization:** To promote cell adhesion to the Au-coated VA-SiNTs and increase the hydrophilicity of the surface, the samples were coated with poly-D-lysine (PDL; Sigma-Aldrich). This was achieved by first immersing the samples in 70% ethanol for sterilization. The samples were then incubated in a laminar flow cabinet to dry for 30 min. Following this, samples were treated with UV/Oz (Cleaner Samco UV) for 30 min before sterilizing for further 5 min under UV light in laminar flow cabinet. Next, 1–2 μL of PDL at the concentration of 167 μg/mL in ultra-pure water was drop-casted onto the pattern area. After placing the samples under vacuum for 10 min, they were incubated at 4 °C for 9 h. Finally, samples were washed extensively (5×) with ultra-pure water and dried in laminar flow cabinet before use. The influence of PDL coating on wettability of the surface was examined through contact angle measurement. Three conditions were investigated: immediately after UV/Oz treatment (Au-only without PDL coating; left), 30 min after UV/Oz treatment (Au-only without PDL coating; middle), and 30 min after UV/Oz treatment (PDL-coated Au-coated; right). As shown in Fig. S3, when the sample (i.e., Au-coated NTs) was treated with UV/Oz for 30 min, it exhibited a hydrophilic surface. Yet after only 30 min of incubation under the laminar flow cabinet for UV sterilisation, the sample became less hydrophilic. In contrast, the PDL coating allowed the surface of the sample to maintain a high level of hydrophilicity after UV sterilisation under the laminar flow cabinet for 30 min.
     2. **Loading of bioactive cargos into Au-coated NTs:** Prior to loading, the PDL-coated sample (Au-coated NTs) was sterilized for 30 min with UV light under a laminar flow cabinet. Next, a PDMS enclosure was placed on the sample, confining the access only to the pattern area (3 mm × 3 mm). For loading, 5 µL of cargo solution was added into the PDMS enclosure, covering the pattern area. In this study, four distinct biomolecules were selected: Goat anti-mouse antibody (IgG-AF647, 200 ng/μL; Life Technologies), Cy5-tagged GFP-encoding mRNA (150 ng/µL; Trilink Biotechnology), Cy3-tagged GFP-encoding plasmid DNA (pDNA, 120 ng/µL; Trilink Biotechnology), and scramble (negative)/anti-TRIOBP siRNAs (10 μM; Life Technologies). All loadings were performed on the orbital shaker to allow uniform insertion of cargos into the NTs. For mRNA, pDNA, and siRNA, the loading was done on ice to avoid degradation of cargos during loading on the orbital shaker. Before imaging with confocal laser microscopy, the PDMS enclosure was carefully peeled-off and the excess cargo solution was removed using an auto pipette. The sample was then rinsed once with Dulbecco's phosphate buffered saline (DPBS; Thermo Fisher Scientific) to remove the unloaded cargos.
     3. **Cell seeding:** In this study mouse embryonic fibroblast cells (GPE-86; ATCC, CRL-9642) cells were chosen. The GPE-86 cells were grown and maintained in complete Dulbecco’s modified Eagle’s medium (DMEM, Gibco) supplemented with 10% fetal bovine serum (FBS, Gibco), 1 mM sodium pyruvate (Gibco), 2 mM L-glutamine (Gibco), 100 U mL^–1^ penicillin (Gibco), and 100 μg mL^–1^ streptomycin (Gibco). For cell seeding, first 70 µL of the cell suspension (0.1 × 10^6^ cells/mL in complete DMEM) was placed inside the PDMS enclosure. The presence of enclosure confined the cells onto the pattern area. The sample was then incubated at 37 ºC with 5% CO_2_ for 3 h to allow the cells to spread and adhere to the PDL-coated NTs. Due to highly hydrophobic nature of PDMS, the enclosure promoted the cells to only attach to the platform and not to the side walls of the PDMS enclosure. After the incubation period, the PDMS enclosure was easily peeled-off using a tweezer.
     4. **Nanoscale-EP via the ENI platform:** To perform nanoscale-EP using the engineered ENI platform, the 33220A Function/Arbitrary Waveform Generator (Agilent Technologies) was used. After 3 h incubation at 37 ºC and 5% CO_2_, the samples were placed in the holder and series of monophasic square-wave pulses were applied to each sample separately (amplitudes 5 V and 10 V; frequency 20 Hz; pulse width 400 μs; number of cycles 600). The nanoscale-EP process was followed by incubation of the samples for 30 min in fresh complete DMEM.
     5. **Preparing cells for analysis post nanoscale-EP:** To analyze the cells post nanoscale-EP, the cells were lifted off from the NT arrays by adding 200 µL of trypsin-EDTA (0.25%) onto the samples. After 2 min incubation at 37 ºC and 5% CO_2_, 300 µL of fresh complete DMEM was added onto the samples to neutralize the trypsin. Once the cells were lifted off, they incubated at 37 ºC and 5% CO_2_ for further analysis.
     6. **Recycling and reusability of the ENI platform:** To confirm the structural integrity of the PDL-coated NTs (Ti/Au-coated) post nanoscale-EP, SEM images were obtained from NTs before and after applying a series of 10 V pulses with 400 μs pulse width for 30 s. The SEM images showed that the Ti/Au coating remained intact, confirming platform stability post nanoscale-EP (Fig. S6). We attributed this largely to the high quality and stability of the Ti layer under the Au layer. This preservation of the NTs and the metal coating post nanoscale-EP allowed for recyclability of the platform. For recycling and reusing the NT samples, they were first immersed in pre-warmed (37 ºC) trypsin-EDTA (0.25%) and incubated at 37 ºC and 5% CO_2_ for 5 min. Next, the samples were rinsed with 70% (v/v) ethanol and ultrapure water, and dried under a nitrogen jet. Post cleaning, the samples were treated with UV/Oz for 30 min to remove any organic residues from the surface and immersed in immersed in 70% (v/v) ethanol and dried under the laminar biosafety cabinet. Next, a new PDL coating was reapplied as described in section 1.3.1. Before each experiment, the samples were placed under UV in the laminar hood for 30 min.

For samples with significant levels of residues from cells post experiment, complete restoration was performed. For this process, the samples were first immersed in piranha (3:1, H_2_SO_4_:H_2_O_2_ v/v) for 10 min. Next, the samples were rinsed with DI water and dried under a nitrogen jet. The samples were then immersed in Au etchant (Gold Etchant TFA; Transene Company Inc.) for 5 min. Next, the samples were thoroughly rinsed with DI water and dried under a nitrogen jet. The samples were then cleaned again with piranha (3:1, H_2_SO_4_:H_2_O_2_ v/v) for 10 min and thoroughly rinsed with DI water and dried under a nitrogen jet. Finally, the samples were loaded into the DC/RF sputtering system (Hummer BC-20 Anatech). At 10^-7^ Torr, first a thin layer of Ti (10 nm) was sputter coated onto the samples. Next, without breaking the vacuum, Au was deposited onto the samples (50 nm).

- 1. **Conventional bulk EP (BEP):** For delivery of Cy5-mRNA-GFP into GPE-86 cells via conventional BEP, we used the Bio-rad Gene Pulser Xcell Electroporation System and Gene Pulser electroporation buffer (Bio-Rad Laboratories, US). For bulk EP two conditions were used. In condition 1, the cells were electroporated without Cy5-mRNA-GFP (negative control (Neg.)). In condition 2, the cells were electroporated with the mRNA at a concentration equivalent to the capacity of the NTs (1.8 ng mRNA/10^6^ cells; equimolar amount converted from that used in ENI for direct comparison). The capacity of NTs in the engineered ENI platform was calculated using: Volume of internal (NT) = πr^2^h; NT inner radius (r) = 150 nm; internal height of cavity within each NT (h) = 1.7 μm. This calculates the volume capacity of each NT to be 0.12 μm^3^.

The ENI platform’s performance was also tested against BEP with the optimized condition, which required 1000-fold higher cargo concentration (1.6 µg mRNA/10^6^ cells; optimized amount of mRNA) compared to the ENI platform (Fig. S9).

To perform bulk EP, the cell suspension in complete DMEM was centrifuged (1300 rpm, 5 min). After removing the supernatant, the EP buffer was added to resuspend the cells. Immediately after, 400 µL of the cell suspension was added to the EP cuvette (0.4 cm gap; Bio-Rad) along with the desired amount of cargo. After placing the cuvette in the electroporator holder, square-wave pulses were applied to the sample (amplitude 200 V; pulse width 8 ms). Post EP, the cells were added into fresh complete DMEM and incubated for 6 h before analysis via flow cytometry.

**1.5. Preparation and maintenance of primary human T cells:** Human primary T cells were obtained from Australian Red Cross Lifeblood from healthy donors (age 20–40 years’ old). Peripheral blood mononuclear cells (PBMCs) were harvested via density gradient centrifugation using Ficoll Paque Plus (Sigma-Aldrich). Red blood cells (RBCs) were removed from PBMCs using 1× RBC lysis buffer (ThermoFisher). Before each experiment, a frozen vial containing the collected PBMCs was thawed. The primary human T cells were isolated and purified from the PBMC collection using the MACS Pan T Cell Isolation Kit (human, Miltenyi Biotec), and were stimulated with anti-human CD3, anti-human CD28, and recombinant human IL-2 (rhIL-2) for 48 h before cell seeding onto the ENI platform. Primary human T cells were grown and maintained in complete RPMI (RPMI-1640 (Gibco), consisting of 10% FBS, 10 × 10^−3^ M 4-(2-hydroxyethyl)-1- piperazineethanesulfonic acid, 1 × nonessential amino acids solution (Gibco), 1 × 10^−3^ M sodium pyruvate, 2 × 10^−3^ M L-glutamine, 100 U mL^–1^ penicillin, 100 μg mL^–1^ streptomycin, and 55 × 10^−6^ M 2-mercaptoethanol (Gibco)) supplemented with rhIL-2 (100 IU mL^–1^, Roche). For pre-activation and expansion, high-binding 96-well-plate coated with anti-CD3 (5 μg mL^–1^) and anti-CD28 (5 μg mL^–1^) were used to stimulate the T cells for 48 h. All cells were incubated at 37 °C with 5% CO_2_.

**1.6 Nanoinjection of pDNAs into primary human T cells via ENI platform:** Here, we have shown the ENI platform’s capacity to nanoinject pDNAs (Cy5-tagged pDNAs encoded for GFP) into hard-to-transfect primary human T cells. For this purpose, we used NT arrays of 3 µm spacing to accommodate for primary human T cells that are smaller in size than GPE-86 mouse fibroblast cells. First, we demonstrated the successful loading of the Cy5-tagged pDNAs into the Au-coated NTs, indicated by the bright fluorescent spots at each NT location (Fig. S10a). After 24 h, the quantification of flow cytometry showed a significantly higher percentage of Cy5^+^ population and therefore a higher pDNA delivery in the ENI-10 V transfected cells (45.9%) compared to the ENI-0 V transfected group (29.1%; Fig. S10b,c). Similarly, when analyzing the GFP expression in the T cells post-treatment, it was evident that the percentage of GFP^+^ population was significantly higher in ENI-10 V transfected cells (18.1%) compared to the ENI-0 V transfected group (4.5%; Fig. S10d, e). Additionally, confocal imaging further confirmed both Cy5 and GFP signals in ENI-10 V transfected T cells (Fig. S10f), suggesting successful delivery and expression of the pDNA constructs in the primary human T cells. It should also be noted that the ENI process (10 V) had negligible impact on the cells’ health, achieving a 90.9% viability.

We also conducted a comparison between the ENI platform and conventional BEP using the Bio-Rad Gene Pulser Xcell from Bio-Rad Laboratories. When the BEP approach was used with an equivalent amount of pDNA to the loading capacity of the NTs within the ENI platform (1.8 ng pDNA/10^6^ cells; direct comparison), it resulted in significantly lower pDNA delivery (approximately 12-fold) and GFP expression (18-fold) compared to the ENI-10 V transfected group. We also considered the optimized concentration of pDNA for BEP (1.6 µg pDNA/10^6^ cells; Optimized), which is 1000-fold higher than the quantity required by the ENI platform. Flow cytometry analysis showed a significantly lower percentage of Cy5^+^ (38.1%) and GFP^+^ (9.1%) population compared to the ENI-10 V transfected group (45.9% for Cy5^+^) and (18.1% for GFP^+^). This comparison serves to highlight the improved performance of the ENI platform over the BEP approach.

1. **Theoretical simulations of electric field across the ENI platform**

The electric field distribution across the platform during the nanoscale-EP was simulated through Ansys (2020 R1) using the Maxwell 2D module. One key assumption considered during electric field modeling was the absence gap at the NT–cell membrane interface. For these simulations, a 2D model of NTs with 2 µm height and 100 nm thick walls was created. The computational domain was set to be a region (20 µm wide) that included four NTs evenly spaced 5 µm apart. The bottom planar electrode and NTs were set as Au and the reference electrode was Pt using ANSYS default material properties. Following the experimental ENI setup, the distance between Au-coated NTs and Pt electrode was set to be 200 µm (Fig. S4a). The region above the cell and within all NTs was assumed to be filled with culture media with a conductivity of 0.5 S/m and a relative permittivity of 80. The cell was designed to be 10 µm wide and 5 µm high, with a membrane 6 nm thick. The cytoplasm conductivity and relative permittivity were 0.2 S/M and 59.9 respectively and 5 × 10^-7^ S/m and 88.1 for the cell membrane. Cells were assumed to conform to the shape of the NTs and be in close contact with the conductive NTs, avoiding presence of media between cell membrane and the NTs. This model resulted in a mesh convergence containing approximately 200,000 elements with additional refinement in the cell membrane region around the top of the NTs (Fig. S4b). The mesh convergence study identified the top corners of the NTs as the region of highest gradient in the electric field strength (E). To accurately measure E in this region, the maximum element size was specified for each region as follows. In the cytoplasm of the cell, the maximum element size was set to 100 nm (Fig. S4bi,ii). In the cell membrane the maximum element size was set to 10 nm (Fig. S4biii). However, in the region near the NT’s rim the maximum element size was set to 0.1 nm (Fig. S4biv). To confirm the concentration of electric field at the NT’s rim, a 2D profile plot of electric field intensity was obtained at the NT’s rim during the nanoscale-EP (5 V applied voltage). As demonstrated in Fig. S5a, the cross-sectional plot of NT’s rim indicated the maximum electric field at the edges of the NTs with highest electric field intensity at the outer edge of the NTs (Fig. S5b).

1. **Additional figures and data**


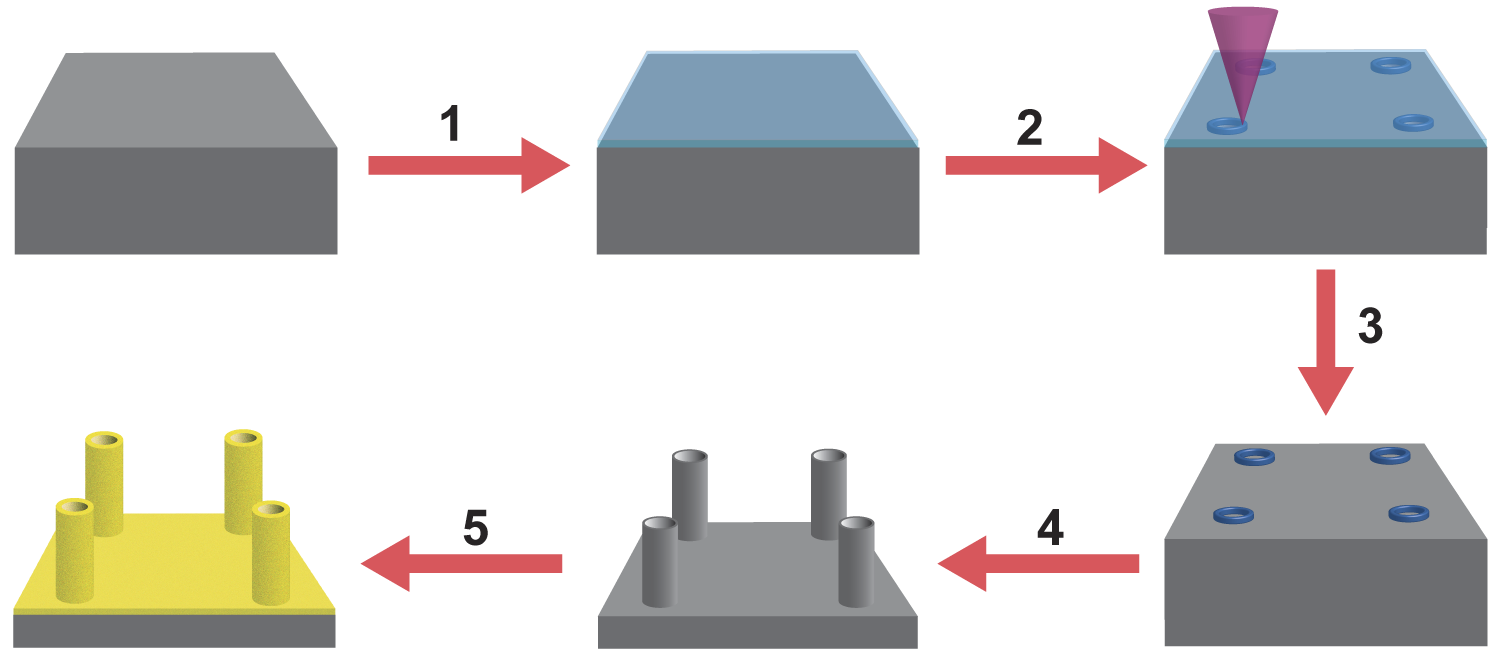


**Scheme S1** Fabrication process of ENI platform’s Au-coated VA-SiNTs. (1) The piranha cleaned Si wafer was coated with a thin layer of electron sensitive resist (HSQ). (2) The resist-coated wafer was exposed by an electron beam to create the desired ring patterns (300 nm inner and 500 nm outer diameter with 5 µm spacing). (3) The sample was chemically developed to remove unexposed resist. (4) Reactive ion etching was used to etch NTs into the Si substrate. (5) The piranha cleaned sample was sputter coated with a thin layer of Ti (~10 nm) and Au (~50 nm).

**
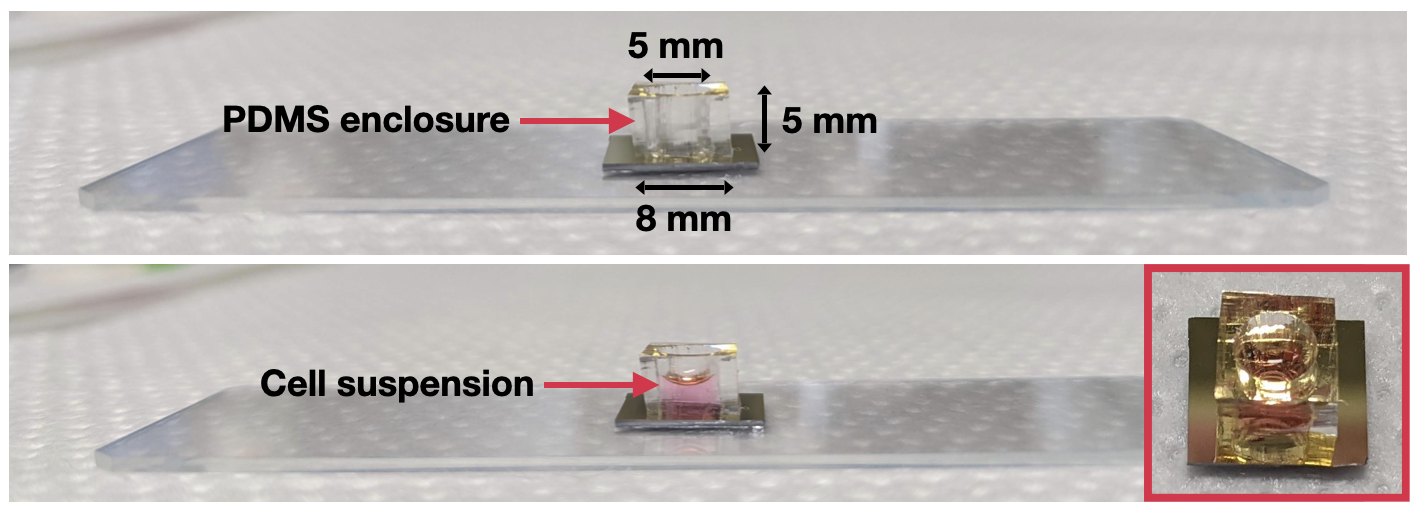
**

**Fig. S1** The biocompatible PDMS enclosure. The enclosure (height = 5 mm, width = 8 mm, opening diameter = 5 mm) consists of an opening consistent with the size of the pattern area (3 mm × 3 mm).


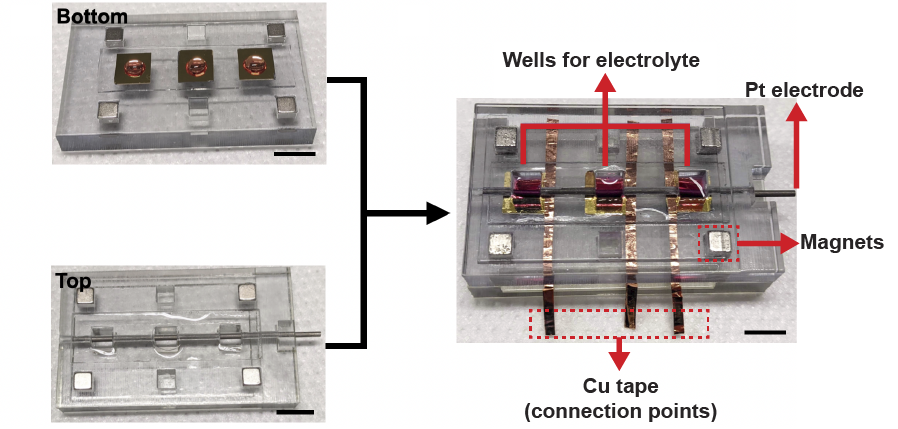


**Fig. S2** The 3D printed biocompatible holder. The two portions of holder are held together with four magnets. The Cu is pressed onto the samples once the holder is put together. The Pt electrode is housed in the top portion of the holder. Scale bars: 10 mm.


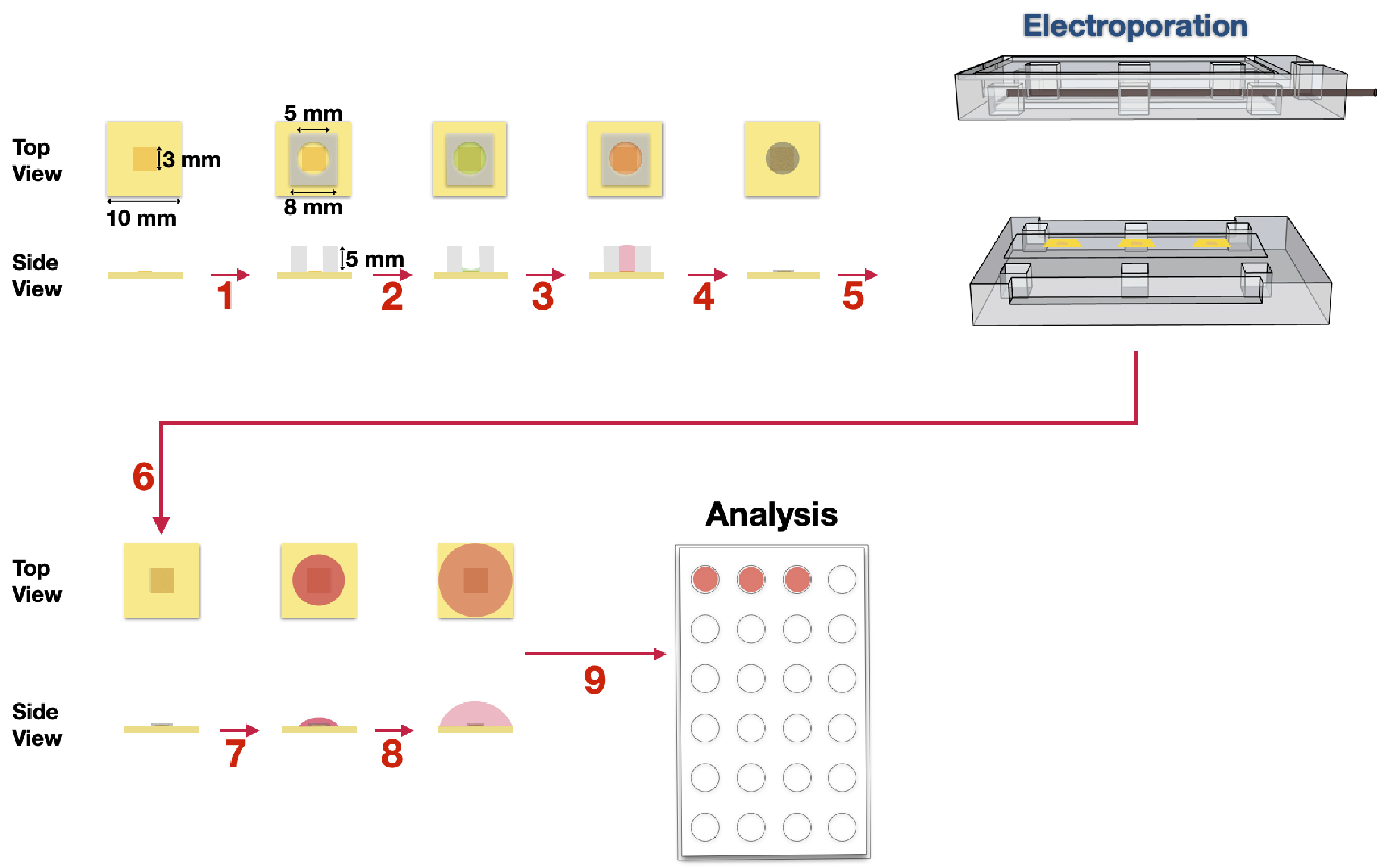


**Scheme S2** The complete process of the ENI platform’s operation. (1) The sterilized PDMS enclosure is placed onto the ENI platform. (2) The cargo solution is added into the PDMS enclosure. (3) The cell suspension in fresh complete DMEM media is added to the PDMS enclosure. (4) Samples are incubated at 37 ºC and 5% CO_2_ for 3 h and the PDMS enclosure is peeled-off after the incubation period. (5) Treatment of samples with the nanoscale-EP by placing the samples inside the holder. (6) The cells outside of the pattern area are removed. (7) Trypsin-EDTA (0.25%) is added onto the pattern area. (8) The samples are washed with 200 μL of fresh culture media and the cells are removed. (9) The extracted cells are analyzed.


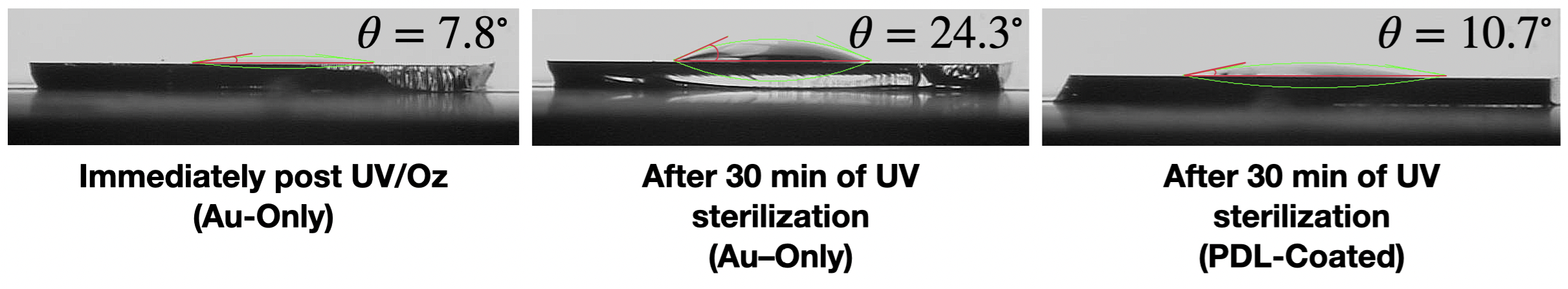


**Fig. S3** Contact angle measurements at the pattern area (3 mm × 3 mm) within the ENI platform. Three conditions were investigated: immediately after UV/Oz treatment (Au-only; left), 30 min after UV sterilization (Au-only; middle), and 30 min after UV sterilization (PDL-coated; right).

**
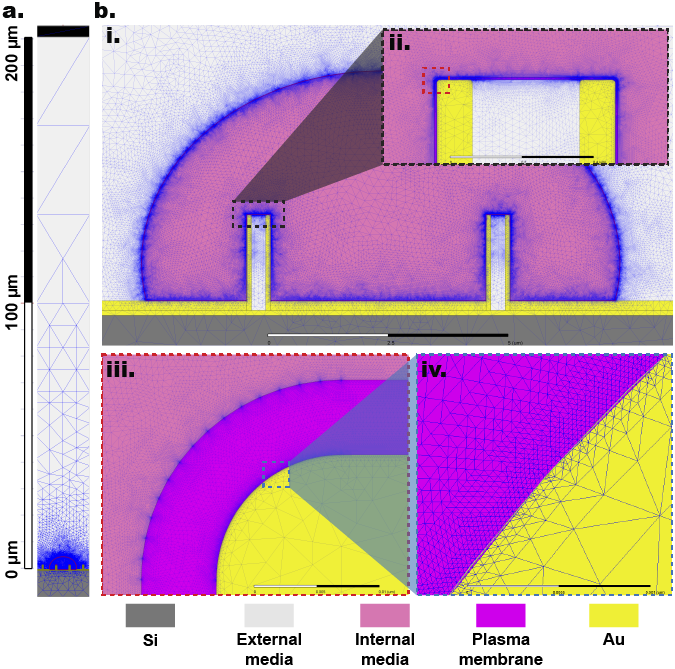
**

**Fig. S4** The mesh convergence model for electric field simulation across the ENI platform during the nanoscale-EP. **a** The overview of mesh convergence model of the entire ENI setup, demonstrating approximately 200,000 elements with refinement at the cell–NTs region. **b** The overview of the mesh convergence model at the cell–NTs region with maximum element size of 100 nm for the cytoplasm of the cell (i, zoom-in view (ii)) and additional mesh convergence refinement at the top corners of the NTs with maximum element size of 10 nm (iii) and 0.1 nm (iv).

**
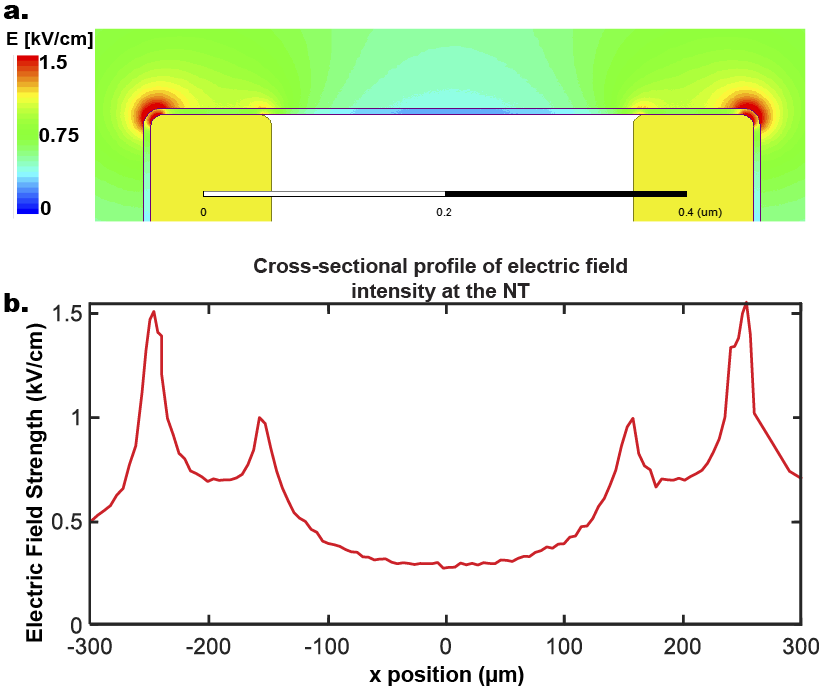
**

**Fig. S5** The 2D electric field profile at the NT’s rim during 5 V applied voltage. **a** The 2D visualization of NT’s cross-sectional profile, showing maximum electric field strength varies over the width of the cell membrane. **b** A 2D electric field intensity plot of NT’s cross-sectional view, showing and confirming the presence of maximum peak intensity at the NT’s edges.


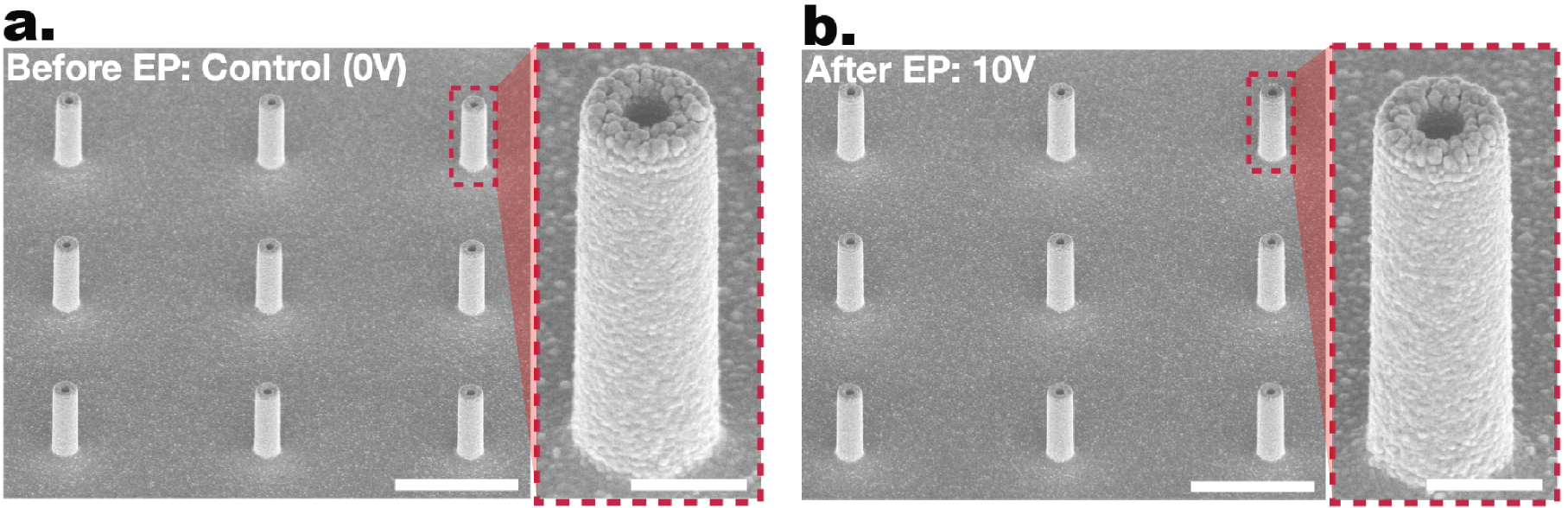


**Fig. S6** Influence of applied voltage (10 V; 20 Hz; 400 µs; 600 cycles) on Au-coated NTs. SEM images showing the comparison between Au-coated NTs before (**a**) and after (**b**) nanoscale-EP. Scale bars: 3 µm and 500 nm (zoom-in).

**
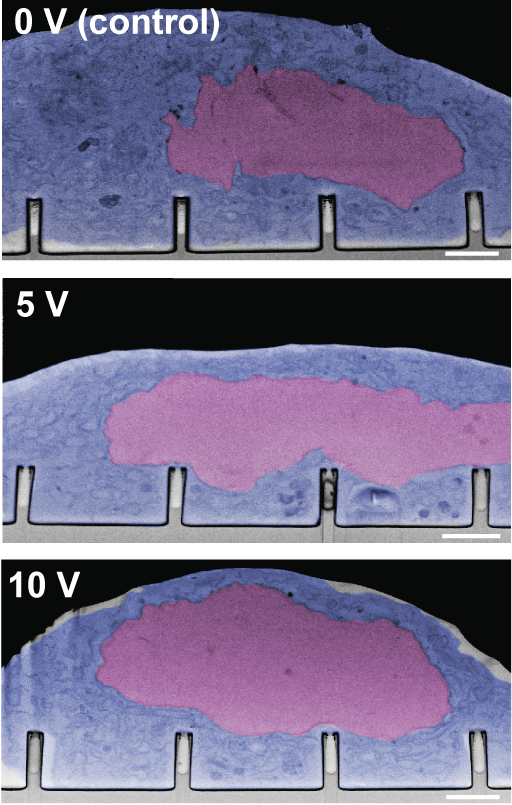
**

**Fig. S7** FIB-SEM images of GPE-86 on Au-coated NTs. The comparison of cell–NT interface before (0 V; control) and after nanoscale-EP (5 and 10 V; 20 Hz; 400 µs; 600 cycles). Scale bars: 2 µm.

**
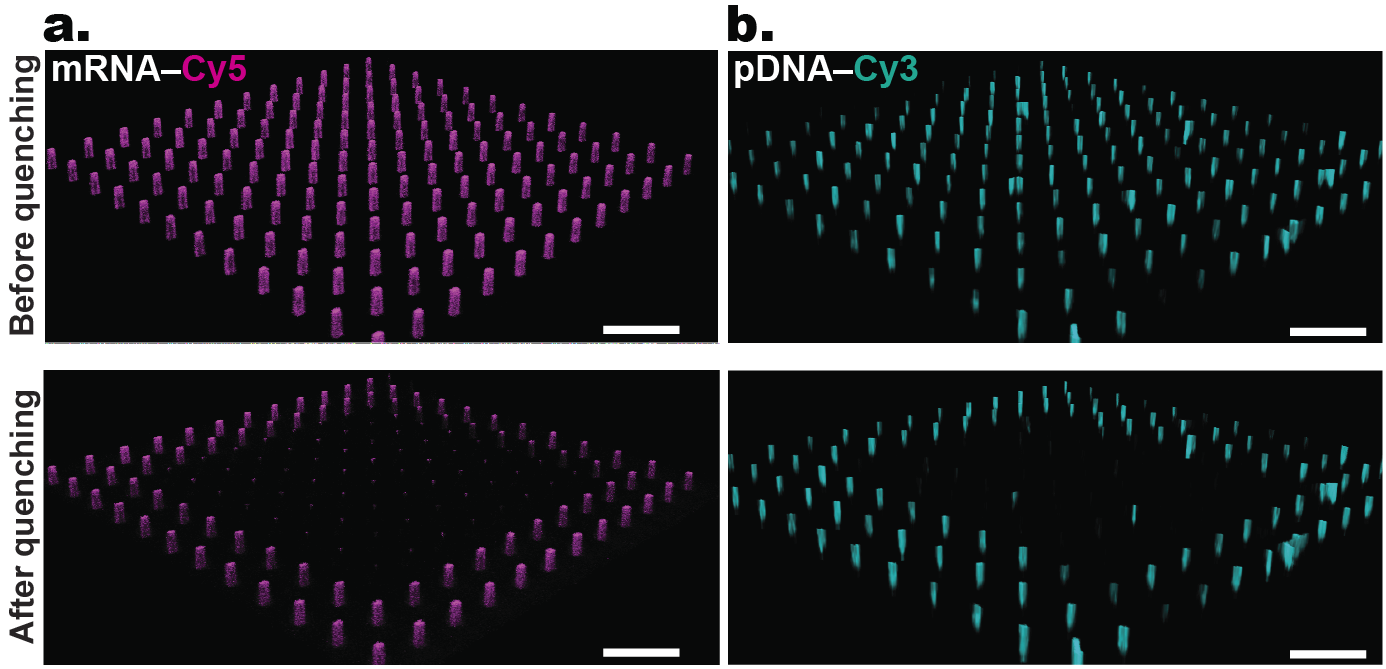
**

**Fig. S8** Loading of the mRNAs and pDNAs into the ENI platform and the confirmation of loading. **a** Loading of the Cy5-tagged GFP-encoding mRNA and the confirmation of true loading by inducing quenching. **b** Loading of the Cy3-tagged GFP-encoding pDNA and the confirmation of true loading by inducing quenching. Scale bars: 10 µm.


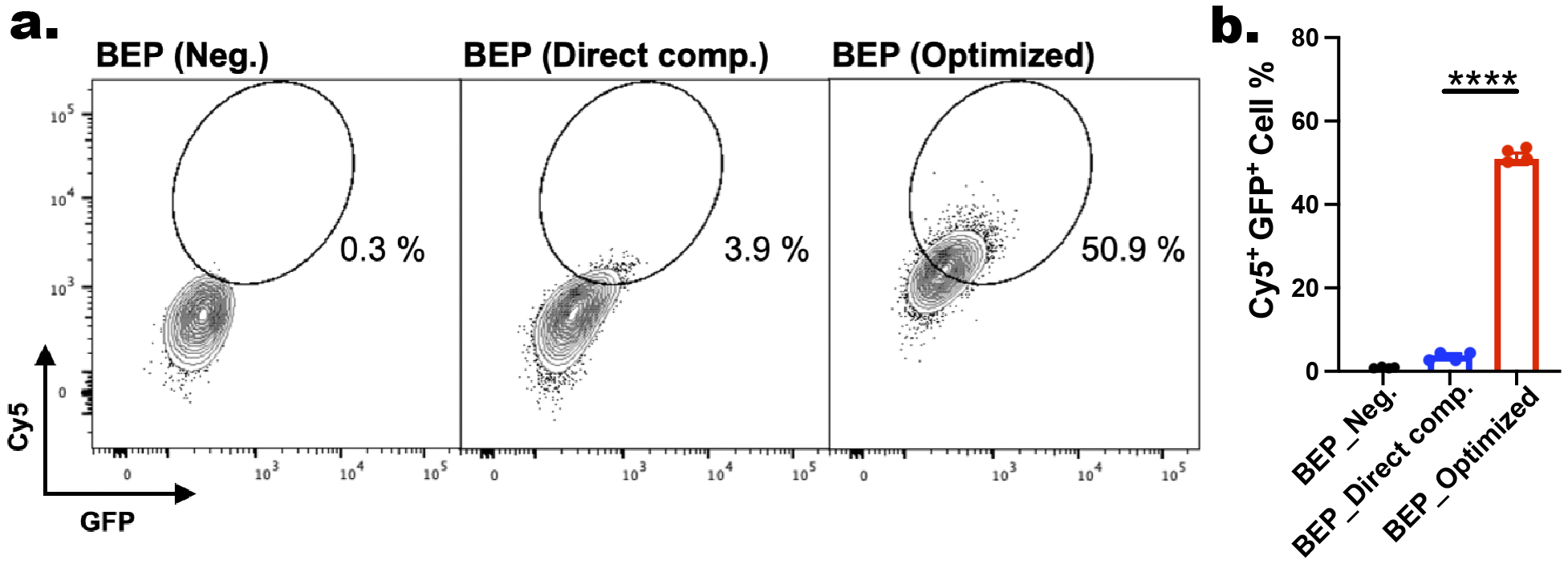


**Fig. S9** Delivery of Cy5-mRNA-GFP into GPE-86 cells through conventional BEP. **a** Flow cytometry analysis of GPE-86 cells processed using BEP with circle gating indicating Cy5^+^GFP^+^ population. **b** Quantification of the percentage of cells showing both Cy5 and GFP signals. Error bars indicate ± SDs, n = 4, ****P < 0.0001, one-way ANOVA.





**Fig. S10** Nanoinjection of Cy5-pDNA-GFP into primary human T cells using the ENI platform. **a** Confocal images showing 3D-views of the loading of Cy5-tagged (magenta) pDNA. Scale bar: 3 µm. **b** Flow cytometry analysis showing the histogram and quantification of fluorescence intensity of Cy5 within T cells from each group. **c** Quantification of the percentage of T cells exhibiting Cy5 signals. Error bars indicate ± SDs, n = 3, ****P < 0.0001, one-way ANOVA. **d** Flow cytometry analysis showing the histogram and quantification of fluorescence intensity of GFP within T cells from each group. **e** Quantification of the percentage of T cells exhibiting GFP signals. Error bars indicate ± SDs, n = 3, ****P < 0.0001, one-way ANOVA. **f** Confocal microscopy images demonstrating the insertion of Cy5-pDNAs and the subsequent GFP expression in the T cells from the ENI-10 V group after 24 h. Scale bars: 10 µm.


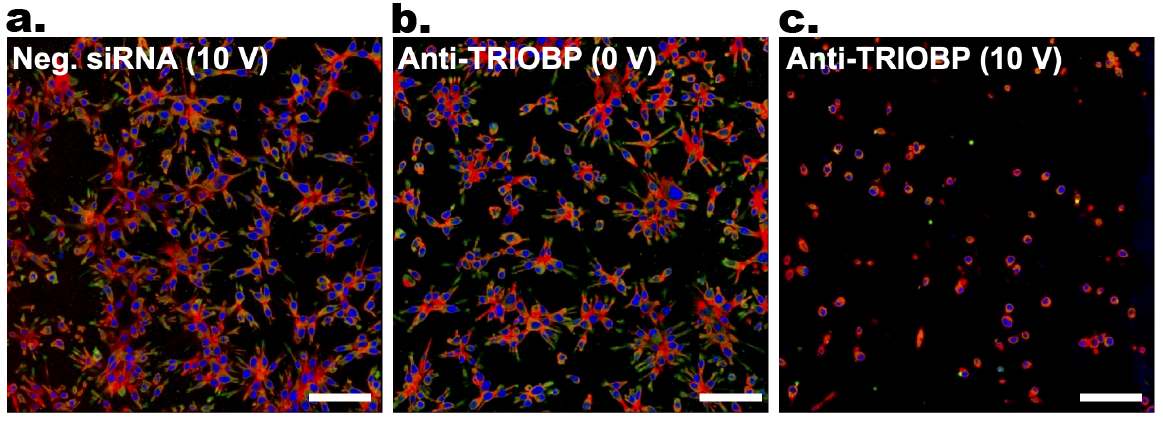


**Fig. S11** Large-scan confocal microscopy images showing the distribution of GPE-86 cells after 24 h culture. **a** Cells on NT arrays loaded with negative control scramble siRNA (Neg. siRNA (10 V)). **b** Cells on NT arrays loaded with TRIOBP-targeting siRNAs (Anti-TRIOBP (0 V)), and **c** Cells on NT arrays loaded with TRIOBP-targeting siRNAs (Anti-TRIOBP (10 V)). Cells were stained with Hoechst (blue), phalloidin (red), and TRIOBP antibody (green). Scale bars: 100 μm.
